# Identification of Neuroactive Chemicals in Crude Oil-Derived Water-Accommodated Fractions

**DOI:** 10.1021/acs.est.5c06859

**Published:** 2025-12-22

**Authors:** Nadia K. Herold, Lisbet So̷rensen, Mari E. Creese, Jasmine Nahrgang, Nicole Schweiger, Tamara Tal

**Affiliations:** 1 Ecotoxicology Department, Chemicals in the Environment Research Section, Helmholtz Centre for Environmental Research − UFZ, Leipzig 04318, Germany; 2 Climate and Environment, 555971 SINTEF Ocean, Trondheim 7465, Norway; 3 Department of Chemistry, Norwegian University of Science and Technology (NTNU), Trondheim 7491, Norway; 4 Department of Arctic and Marine Biology, UiT The Arctic University of Norway, Tromsø 9037, Norway; 5 Medical Faculty, Leipzig University, Leipzig 04103, Germany

**Keywords:** water-accommodated fraction, neuroactivity, zebrafish, behavioral phenotyping, effect-directed analysis

## Abstract

Crude oil-derived water-accommodated fractions (WAFs) are complex mixtures containing bioavailable constituents. We applied an automated zebrafish behavior-based assay to assess potential neuroactivity following exposure to WAF and chemical fractions including polycyclic aromatic hydrocarbons (PAHs), resin, naphthalene (NAP), monoaromatic hydrocarbon (MAH), and saturate fractions. Zebrafish larvae were exposed to WAF concentrations (9.8–100%, 1 g/L loading) and assessed using the 26-end point visual and acoustic motor response assay at 5 day postfertilization. WAF exposure elicited concentration-dependent behavioral effects, including reduced dark-period activity, impaired acoustic startle responses, and inappropriate activity during light and typically quiescent interstimulus periods. All WAF fractions were neuroactive, eliciting dark-phase hypoactivity and fraction-specific hyperactivity patterns. Exposure to PAH, MAH, or NAP fractions also impaired habituation learning. Comprehensive two-dimensional gas chromatography–mass spectrometry identified phenanthrene, 2-methylanthracene, and ethyl 4-ethoxybenzoate as the dominant PAHs. In contrast, the resin fraction was chemically diverse. Behavioral profiles of zebrafish exposed to PAH fraction constituents or an artificial mixture recapitulated WAF-like effects. Neurobehavioral fingerprinting predicted that WAF neuroactivity may arise from structurally diverse chemical classes disrupting multiple molecular targets, including disruption of peroxisome proliferator-activated receptor delta- or gamma-aminobutyric acid type A receptor-dependent signaling. Taken together, integration of chemical fractionation, high-resolution behavior phenotyping, and mode-of-action fingerprinting supports mechanistic dissection of environmentally relevant mixtures.

## Graphical abstract

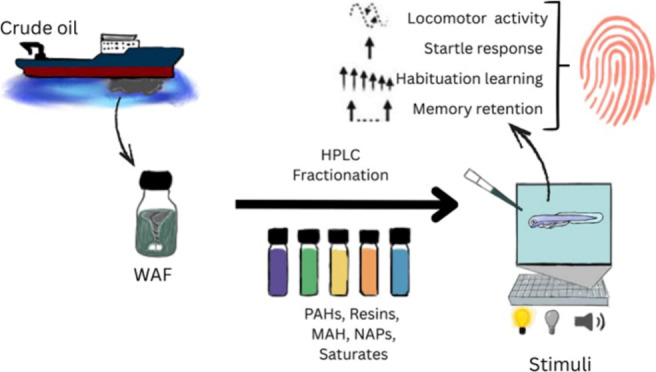

## 1 Introduction

The continuing use and transportation of petroleum products poses a persistent risk of crude oil spills that endanger aquatic ecosystems.^1,2^ Petroleum hydrocarbons and their derivatives can form complex mixtures with ecotoxicological impacts.^3,4^ The water-accommodated fraction (WAF), comprised of water-soluble oil components, forms by natural weathering and contains PAHs, oxygenated hydrocarbons, and other constituents.^5–8^ The ecological effects of crude oil pollution are extensive, impacting individuals, populations, and ecosystems across multiple trophic levels.^9–12^ Among aquatic vertebrates, fish are particularly sensitive to crude oil exposure, where exposure has been linked to cardiovascular malformations, developmental toxicity, and endocrine disruption.^13,14^ Exposure to PAH-rich WAF fractions causes defective heart looping, pericardial edema, and circulatory collapse in fish embryos.^15–17^ WAF exposure has also been reported to decrease fecundity and alter gametogenesis via endocrine disruption.^12,18^ Exposure to WAF-associated chemicals, particularly PAHs, has also been linked to disrupted neurodevelopment.^19^

While prior research has identified neuroactive candidates that contribute to WAF effects on swimming behavior, most studies focused on a restricted number of well-characterized compounds, often due to analytical challenges in detecting less abundant but potentially potent neuroactive constituents, and employed simplified behavioral assays. To address this data gap, Effect-Directed Analysis (EDA) has emerged as a powerful approach to systematically isolate subsets of key chemicals within complex, environmentally relevant mixtures.^20^ This approach is particularly relevant for crude oil WAFs, whose multiple chemical classes may interact to alter neural function and behavior. To investigate the mechanistic basis of WAF-induced neurotoxicity in early vertebrate development, we combined EDA with a novel high-content behavior assay^21,22^ that returns activity data across 26 end points, including learning and memory-related end points. This integrative strategy enabled component resolved investigation of WAF effects, focusing on the neuroactive potential of WAF fractions. By aligning behavior-based signatures with a curated reference library of neuroactive compounds, this study advances a mechanistically informative approach for identifying toxicologically relevant drivers within complex environmental mixtures.

## 2 Materials and Methods

### 2.1 Zebrafish Husbandry

Zebrafish (*Danio rerio*) from a WIK × UFZ-OBI outcross line were maintained as described previously^21^ under standard laboratory conditions (see the Supporting Information, Section S1), approved by the Landesdirektion Sachsen (24–5131/252/7).

### 2.2 Crude Oil and Preparation of WAF

Crude oil–Water-Accommodated Fraction (WAF) was prepared at using a Goliat Kobbe crude oil 250+ distillation residue (provided by SINTEF Ocean), representing artificially weathered crude oil that simulates oil residue after evaporative loss upon 2–5 days at sea.^23^ This WAF was used for chemical extraction and fractionation for downstream compositional analysis and behavior testing. For zebrafish exposure experiments, WAF was freshly prepared in accordance with Chemical Response to Oil Spills: Ecological Research Forum (CROSERF) protocols.^24^ Specifically, 240 mL Hanks’ Balanced Salt Solution (HBSS) was placed in a magnetic stir bar glass with a flask. The flask was placed on a magnetic stirrer in an incubator at 28 °C with continuous mixing, ensuring no vortex formation. Crude oil (32.2 μL, Goliat Kobbe, density ∼0.75 g mL^–1^) was gently layered over the surface of 240 mL of HBSS to achieve a nominal oil-to-water loading rate of 100 mg/L (corresponding to approximately 24 mg of oil). This loading rate was selected based on range-finding tests performed at 1 g/L and 100 mg/L, with the latter chosen to generate a WAF that elicited behavioral effects without inducing mortality. The system was maintained under constant stirring for 48 h to allow partitioning of the water-soluble hydrocarbons into the aqueous phase after which the WAF was collected and immediately used. Serial WAF dilutions were prepared in 10% HBSS where the highest concentration, referred to as ‘total WAF’ corresponded to 100 mg/L, with subsequent dilutions producing final test concentrations of 100%, 75%, 56.6%, 31.36%, 17.56%, and 9.83% WAF. These concentrations were selected to simulate acute exposure conditions relevant to high-contamination locations of oil spills, where hydrocarbon concentrations may temporarily exceed 10–100 mg/L in near-field contamination events.^25^

### 2.3 WAF Fractionation

To prepare fractions, approximately 20 L of a 1 g/L WAF was prepared by layering the same batch of crude oil residue onto reverse osmosis water (pH 7, conductivity 1000 μS) at a 1 g/L oil-to-water ratio. The mixture was stirred gently for 48 h at 28 °C with 20% headspace. The WAF was collected from the bottom of the aspirator bottles without disturbing the oil layer and ∼17 L was serially extracted with dichloromethane (DCM) as previously described.^26^ Extract volumes were combined, and DCM was evaporated and replaced with *n*-hexane. The volume was adjusted to 2 mL under gentle evaporation using a Turbovap. Half (2*0.5 mL) of the WAF extract was injected serially on an Agilent 1260 series using high-performance liquid chromatography (HPLC) system equipped with a 1260 FC-AS fraction collector and an Agilent 1200 series G1315 diode array (DAD) detector. The system was set up with two μBondapak NH2 10 μm 3.9*300 mm columns in series with backflush. Elution was first performed with *n*-hexane (1 mL/min until 8.8 min, then 2 mL/min until 30 min), followed by back-flushing with DCM (30–40 min).

Based on published protocols for polarity separation of crude oils,^27,28^ injection of a model oil ‘SINTEF mix’^29^ and analysis of obtained discrete fractions were used to optimize WAF fractionation. The cutoff between saturates and aromatics was set before elution of monoaromatic compounds. Fractions were nominally distinguished as “Saturates” (0–8 min), “Monoaromatics” (8–10 min), “Naphthalenes” (10–12 min), “PAHs” (12–30 min), and “Resins” (30–40 min). The resulting chromatograms were monitored by UV (210, 254, and 280 nm). Fractions from duplicate injections were combined into their respective classes, gently evaporated, and reconstituted in *n*-hexane (2 mL). 200 μL were subsampled for characterization. The remaining 1 mL of the WAF extract was diluted to 2 mL. From each fraction and the WAF extract, 900 μL were subsampled and 250 μL dimethyl sulfoxide (DMSO, BioUltra for molecular biology, > 99.5% purity, Sigma-Aldrich) was added and evaporated. The remaining WAF extract (1 mL) was similarly treated. A procedural blank DCM volume similar to that used for extraction of the WAF was prepared and subjected to the same fractionation pipeline as described above. Subsamples of each WAF fraction and blank fraction extracts were diluted (20x and 250x) for chemical analysis.

### 2.4 Chemical Preparation

To test individual chemicals, 20 mM stock solutions were generated by dissolving neat chemicals (≥96.75% purity) in anhydrous dimethyl sulfoxide (DMSO; Sigma-Aldrich). Stock solutions were aliquoted and stored at −80 °C. For each experiment, 250X working solutions were generated by thawing single-use aliquots and performing quarter-log serial dilutions in DMSO within a 96-well polycarbonate square-well microtiter plate, resulting in a final concentration range of 4.4–80 μM except for phenalen-1-one, which was tested between 1.87 and 25.08 μM, as concentrations above this threshold resulted in mortality.

A chemically defined artificial mixture was prepared using the three most abundant PAH fraction constituents identified via chemical analysis (phenanthrene (66.4% of the mixture), 2-methylanthracene (19.4%), and ethyl 4-ethoxybenzoate (14.3%). The compounds were combined in a 500 μL DMSO stock solution to achieve final concentrations reflecting their proportional abundance in the PAH fraction. Specifically, the final stock contained phenanthrene (331.9 μL), 2-methylanthracene (96.8 μL), and ethyl 4-ethoxybenzoate (71.3 μL), resulting in a total combined molarity of 20 mM. The resulting stock was serially diluted in DMSO following the same dilution protocol as described above, yielding 250X working solutions ranging from 0.2 to 80 μM in final test concentrations. This range was tested to explore the concentration window that most closely reproduced the PAH fraction’s behavioral fingerprint.

### 2.5 Zebrafish Exposures

On day 0, zebrafish embryos were bleached for 5 min using 0.05% NaOCl prepared in 10% HBSS then rinsed three times in 10% HBSS. From 0 to 4 days postfertilization (dpf), bleached embryos were maintained in glass crystallization dishes at a density of 50 embryos per 100 mL of 10% HBSS at 28 °C under a 14:10 h light-dark cycle. At 4 dpf, larvae were individually transferred into single wells of a 96-square well polystyrene plate (Uniplate, Whatman microplate devices), each containing 400 μL of 10% HBSS. To reduce evaporation, plates were covered with Microsealers (Bio-Rad) and sealed with parafilm. At 5 dpf, microtiter plates were placed in dark conditions at 28 °C. Zebrafish larvae were exposed to WAF, WAF fractions, individual chemicals, a PAH chemical mixture, or the solvent control (0.4% DMSO) 100 min before the behavior test in the dark under red light. No behavioral effects were observed in larvae exposed to 0.4% DMSO alone. 40 min before the test began, plates were moved into light conditions, in an incubator set to 28 °C. Following the behavior test, zebrafish larvae were visually examined for general malformations, including edemas, body axis defects, and swim bladder inflation status, and lethality. Larvae that were dead, malformed, or had uninflated swim bladders were excluded from behavior analysis. At the conclusion of each experiment, larvae were anesthetized via rapid chilling in ice-cold 10% HBSS then euthanized by freezing.

For WAF fractions, each fraction was assayed at 100% concentration, and compared to its corresponding blank control, prepared as previously described. Exposure solutions were reconstituted by preparing the fraction extracts in 10% HBSS. Larvae were exposed individually in 96-well microplates in which each well received 400 μL of the corresponding test solution. During initial testing, acute mortality was observed in zebrafish larvae exposed to the Resin and WAF Fraction at 100%. To mitigate toxicity while still evaluating behavioral effects, these fractions were retested at lower concentrations of 56%, 31%, and 5.9%. Since larvae exposed to 56% and 31% of the WAF fraction exhibited suppressed motor activity, 5.9% concentration was selected for further behavioral assessments.

### 2.6 Automated Behavior Testing

Larval zebrafish were evaluated using a previously established acoustic motor response (VAMR) assay,^21^ incorporating visual (light, dark) and acoustic (300 Hz, 65/75 dB) stimuli (see the Supporting Information, Section S2 for stimulus schedule).

### 2.7 Processing of Automated Behavior Data

Video tracking was conducted at 25 frames per s using ZebraLab software (ViewPoint) in quantization mode. Raw locomotor activity (Δpixel/sec) was processed using KNIME (v4.6.3) and R (v4.3.2), according to our previous work.^21,22^ Behavioral end points were defined to capture visual and acoustic responses, including the visual startle responses (VSRB, VSR1, VSR2), visual motor responses (VMR1–VMR5), acoustic startle responses (ASR1, ASR2, ASR3), interstimulus intervals (ISI1–3), interend point intervals (IEI1–3), acoustic habituation (ASH1), potentiation of habituation (ASH1/5), acoustic habituation sum (ASHsum), interbout interval (IBI), and memory retention (ASR2/3). Visual startle responses were measured over one second following light-to-dark or dark-to-light transitions. Visual motor responses were calculated as the mean motor activity across subdivided time intervals during the corresponding light or dark phases. Acoustic startle responses were quantified as the mean motor activity across five consecutive low- or high-intensity acoustic stimuli, each one second in duration and separated by one-min ISIs. Motor activity during ISIs and IEIs was calculated as the sum of the activity within each respective period. Habituation metrics were derived from a subset of motor activity during five bouts of thirty high-intensity acoustic stimuli. The acoustic startle habituation (ASH1) was calculated as the relative proportion of motor activity during the final ten taps of the first habituation bout compared to the summed activity of the initial and final ten taps. Potential of habituation (ASH1/5) was determined by calculating the ratio of motor activity during the final habituation bout (ASH5) to the sum of the activities in both the initial (ASH1) and final (ASH5) bouts. The sum of activity across all five habituation bouts was calculated (ASHsum). The inter-bout (IBI) endpoint was computed as the sum of motor activity between habituation bouts. Memory retention (ASR2/3) was calculated as the relative proportion of activity between the ASR2 endpoint and the combined mean responses before (ASR2) and after (ASR3) habituation training. Motor activity for all end points was quantified as changes in pixel intensity per second.

### 2.8 GC×GC-MS Analysis

Comprehensive two-dimensional gas chromatography–mass spectrometry (GC×GC-MS) analyses were performed using a 7890B GC coupled with a 7250 quadrupole time-of-flight mass spectrometer interfaced with a Zoex ZX2 cryogenic modulator. The first-dimension column was a Zebron ZB-1plus (30 m × 0.25 mm × 0.25 μm), and the second-dimension column was a BPX50 (1.0 m × 0.25 mm × 0.25 μm), interfaced by a 1 m × 0.25 mm deactivated fused silica modulation loop. The carrier gas was high purity helium at a constant flow of 1.1 mL/min. Samples (1 μL) were injected at 250 °C splitless. The oven temperature was kept at 60 °C (1 min hold) and ramped at 5 °C/min to 300 °C (10 min hold). The hot jet was offset at +10 °C (1 min hold) and ramped at 7 °C/min to 360 °C (10 min hold). The modulation time was 6 s with a 350 ms pulse length. The transfer line temperature was 300 °C, the ion source temperature was 200 °C, and the quadrupole temperature was 150 °C. The electron ionization (EI) source was operated at 70 eV. The scan speed was 50 Hz, and the recorded mass range was 50–650 mass-to-charge ratio (*m*/*z*). GCImage (version 2.7r3) was used for manual inspection of chromatograms.

Masshunter Unknowns Analysis software was applied to raw data files for deconvolution and tentative identification of analytes via searches against the NIST23.L library, returning the best match with a mass spectral similarity exceeding 80%. Output files containing all detected peaks and assigned tentative identities were exported in.csv format for further processing in R.^30^ To account for peak tailing and slight retention time shifts, peaks were binned if they occurred within a [0.2 min, 0.2 s] retention position quadrant interval and shared identical CAS numbers.

The same GC×GC-MS full scan analysis was performed on all samples, *n*-alkanes, and a suite of organic compounds with varying physicochemical properties (Table S1). For every ten sample injections, a quality assurance sample containing the compounds listed in Table S1 was analyzed, followed by a solvent blank to avoid carryover to real samples. Instrument inlet maintenance, including liner and septum replacement, was conducted every 50 injections, and mass calibration was performed between each sample injection. One-dimensional.csv files containing total ion chromatogram (TIC) responses were extracted from all data files, and second-dimension retention times were calculated in R for each time point based on relative positioning to baseline signal peaks, forcing the baseline to a null position. First-dimension retention indices (RIs) were calculated for all spiked compounds and tentatively identified compounds according to the method of van den Dool and Kratz.^31^

A total of 119 known compounds (Table S1) were used to train (100 compounds) and test (19 compounds) a partial least-squares (PLS)^32–34^ regression model using the R package PLS^31^ to independently predict first-dimension retention indices (RIs) and second-dimension retention times (RTs) based on physicochemical properties extracted from PubChem, including molecular weight, xlogP, TPSA, complexity, atom stereo count, hydrogen bond donor and acceptor counts, rotatable bond count, heavy atom count, defined and undefined atom stereo counts. The model was subsequently used to predict RIs and 2D RTs of tentatively identified compounds in the samples. Deviations between predicted and measured first-dimension RIs and second-dimension RTs were used to filter out unlikely matches, retaining only tentatively identified compounds with deviations of less than 200 RI units and less than 2 s absolute RT difference. Manual investigation of the data set was then performed to assign chemical class identities to the identified peaks.

### 2.9 Data and Code

All underlying data and code can be accessed at https://data.mendeley.com/datasets/8hmr85pjw7/3.

## 3 Results

### 3.1 WAF Is Neuroactive in the Zebrafish VAMR Assay

To evaluate the neuroactive potential of WAF, 5 dpf zebrafish larvae were exposed to 9.83–100% WAF and assessed in the VAMR assay.^21,22^ Behavior responses displayed concentration-dependent disruptions across multiple end points and assay phases (Figure 1 and Figure S1). Acoustic startle responses were the most sensitive end points (ASR1–3), with activity decreasing consistently at all WAF concentrations. At ≥31.4% WAF, a concentration-dependent reduction in VSR1 and baseline activity (BSL3–4) were observed. At higher concentrations, increased activity during the light and dark phases (VMR1–2) and interstimulus intervals (ISI, IEI) emerged. At 100% WAF, a range of end points were disrupted with opposite directionality characterized by VMR1 ISI hyperactivity and reduced activity in acoustic startle responses and dark phase locomotor activity (VMR2–5 and BSL periods). In addition, movement during the habituation period (ASHsum) was significantly impaired (Figure 1b–d and Figure S1).

**Figure 1:**
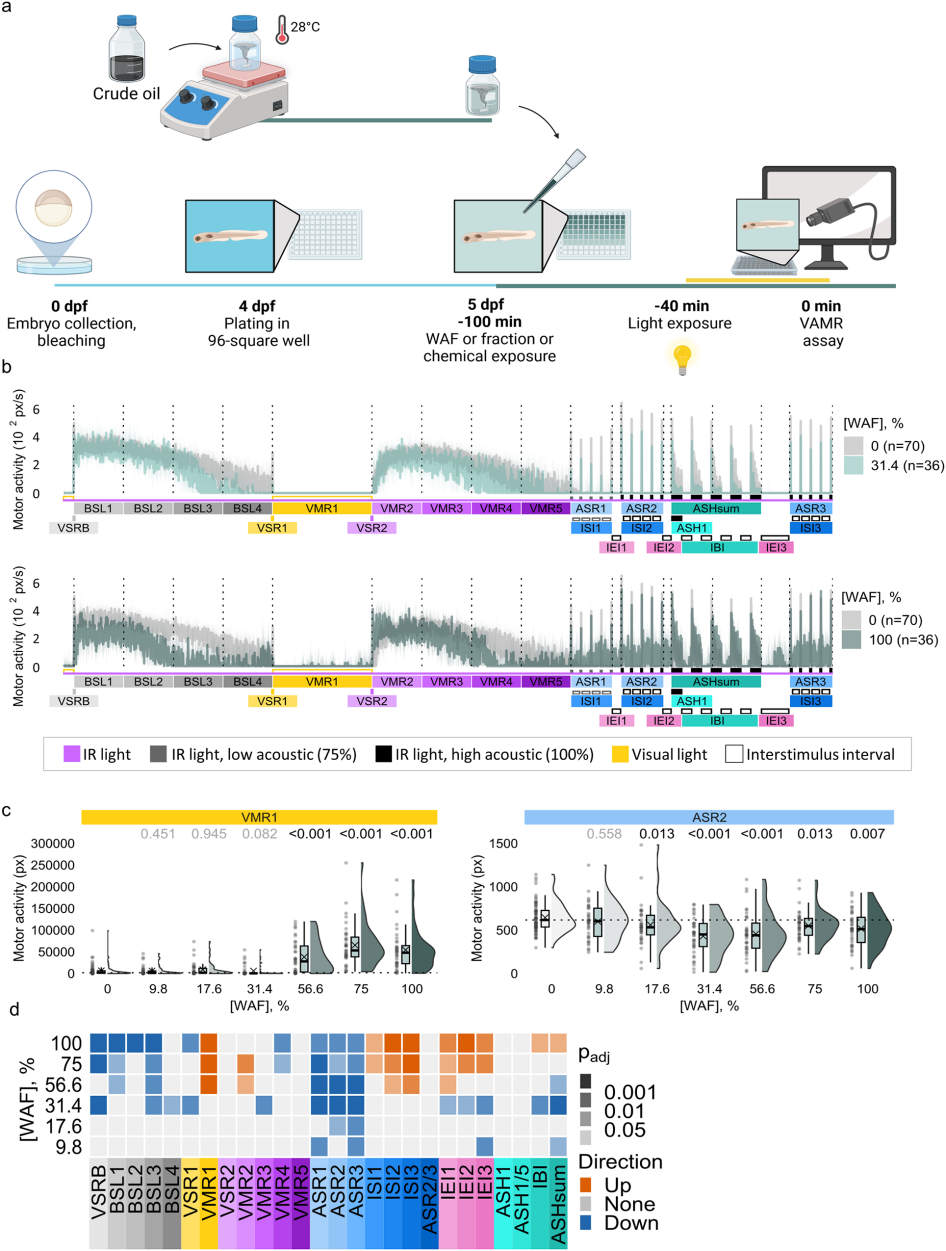
Behavioral responses in zebrafish larvae exposed to crude oil WAF. (a) Experimental design. (b) Motor activity across 26 end points after WAF exposure (31.4%, 100%; teal); control in gray (*n* = 70). (c) Raincloud plots showing motor activity at key end points (VMR1, ASR2); dotted line = control median; adjusted p-values shown. (d) Heatmap depicts statistical differences in motor activity across end points. Orange = increase, blue = decrease, gray = no change (*n* = 36). ASH, acoustic startle habituation; ASR, acoustic startle response; BSL, baseline motor activity; IBI, interbout interval; IEI, interend points interval; ISI, interstimulus interval; WAF, Water-Accommodated Fractions; VAMR, visual and acoustic motor response; dpf, days post fertilization; VMR, visual motor response; VSR, visual startle response; VSRB, visual startle response baseline.

### 3.2 WAF Fractions Are Neuroactive in Early Life-Stage Zebrafish

Crude oil WAF contains a complex mixture of hydrocarbons and heteroatom-containing compounds. To identify chemical classes that contribute to neurobehavioral disruption, WAF was fractionated into five groups: PAH, resin-like compounds (Resin), Saturates, MAH, and NAP. Exposure to all fractions altered neuroactivity profiles. Dark-phase hypoactivity was observed in all exposure groups (Figure 2b–d and Figure S2a–f). Consistent with total WAF exposures (Figure 1b), exposure to the WAF fraction induced hypoactivity in ASR2, VSRB, VSR1–2, and dark-phase end points (BSL1–3, VMR2–4) and hyperactivity in light-phase (VMR1) and interstimulus intervals (ISI2–3, IEI2–3) (Figure 2d and Figure S2a).

**Figure 2:**
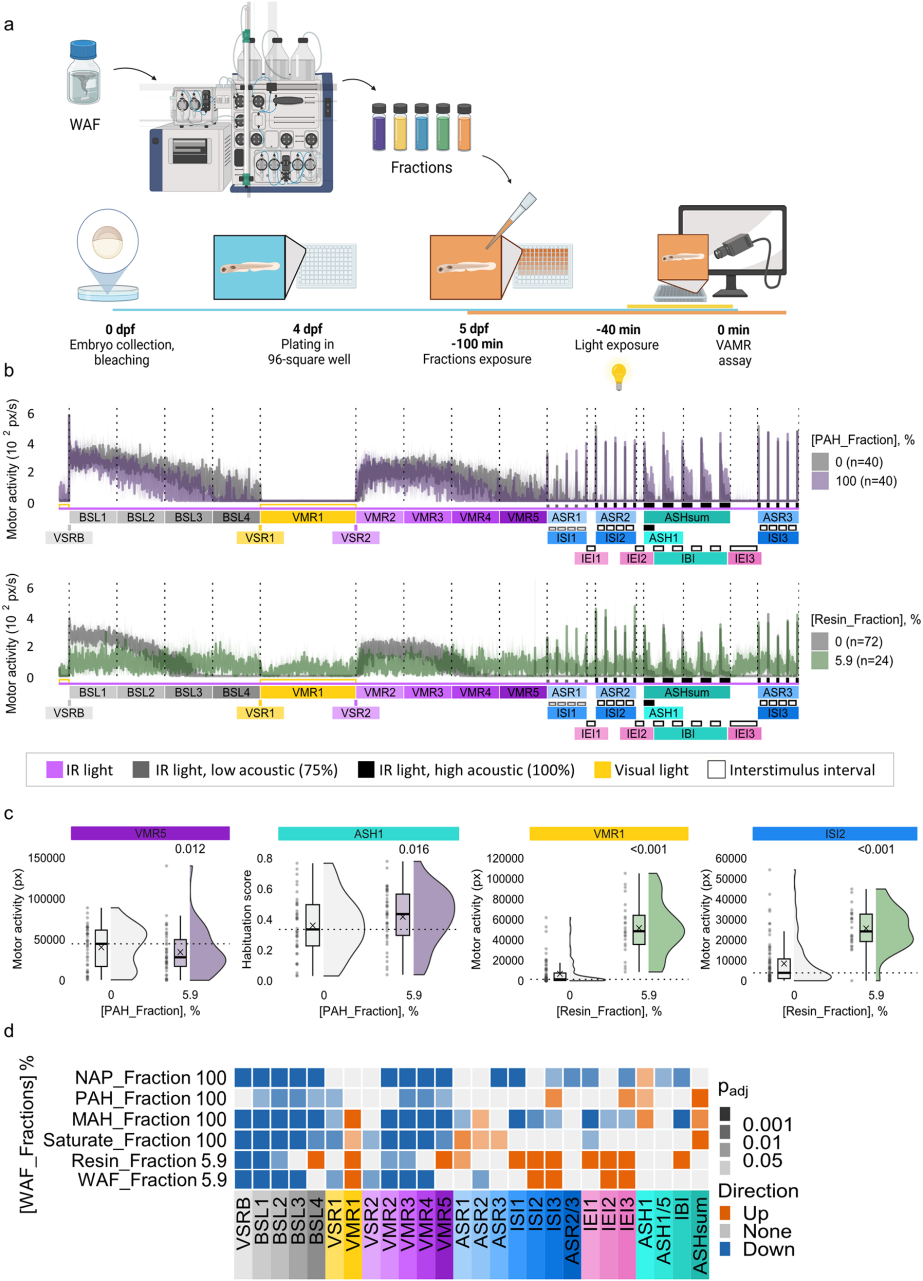
Exposure to WAF fractions alters zebrafish behavior. (a) Experimental design. (b) Motor activity profiles across 26 end points following exposure to PAH (100%, purple), or Resin (5.9%, green) fractions with the control group, exposed to a blank fraction, depicted in gray (*n* = 72). (c) Raincloud plots of selected end points for PAH, and Resin fractions at key end points (VMR5, ASH1, VMR1, ISI2). Dotted line shows control median; adjusted p-values from bootstrap test. (d) Heatmap showing median responses across 26 end points; orange = increase, blue = decrease, gray = no change (*n* = 24–40).

Exposure to the Resin fraction (5.9%) was marked by persistent, inappropriate hyperactivity across all assay phases, including VMR1 and interstimulus periods (ISI, IEI, IBI) (Figure 2b–d and Figure S2b). Exposure to the Saturate fraction elicited dark-phase hypoactivity and light-phase hyperactivity, while uniquely enhancing acoustic startle responses (Figure 2d and Figure S2d). Exposure to the MAH fraction reduced activity in BSL1–4, VMR2–5, VSRB, VSR1, ASR1, and ISI end points, but also triggered light-phase hyperactivity (VMR1), elevated ASR2, and impaired habituation learning (Figure 2d and Figure S2e). Exposure to the PAH fraction evoked WAF-like effects (Figure 2b–d vs Figure 1b,c) characterized by dark-phase hypoactivity and habituation deficits (Figure 2b–d and Figures S2c and S3a). Exposure to the NAP fraction reduced activity in dark-phase end points, acoustic startle (ASR3) and interbout and interstimulus intervals and accelerated habituation learning (ASH1) (Figure 2d and Figures S2f and S3b). Hierarchical clustering showed that profiled derived from exposure to the PAH or Resin fractions resembled the behavioral signature observed upon exposure to the total WAF (Figure S3a).

### 3.3 Fraction-Specific Chemical Profiles

Given that exposure to both WAF or all WAF fractions elicited neurobehavioral disruptions across multiple end points, we next characterized their chemical composition to elucidate potential drivers of these effects. GC×GC-MS analysis revealed distinct compositional profiles for the WAF and its constituent fractions (Figure 3a,b). As predicted, the total WAF contained the highest total chemical abundance, comprising a complex mixture dominated by aromatics, including notably PAHs, and naphthalenes, and oxygenated hydrocarbons. Resin, Saturate, and MAH fractions contained the next highest total peak abundance, with Resin and Saturate fractions dominating by both peak number and intensity.

**Figure 3:**
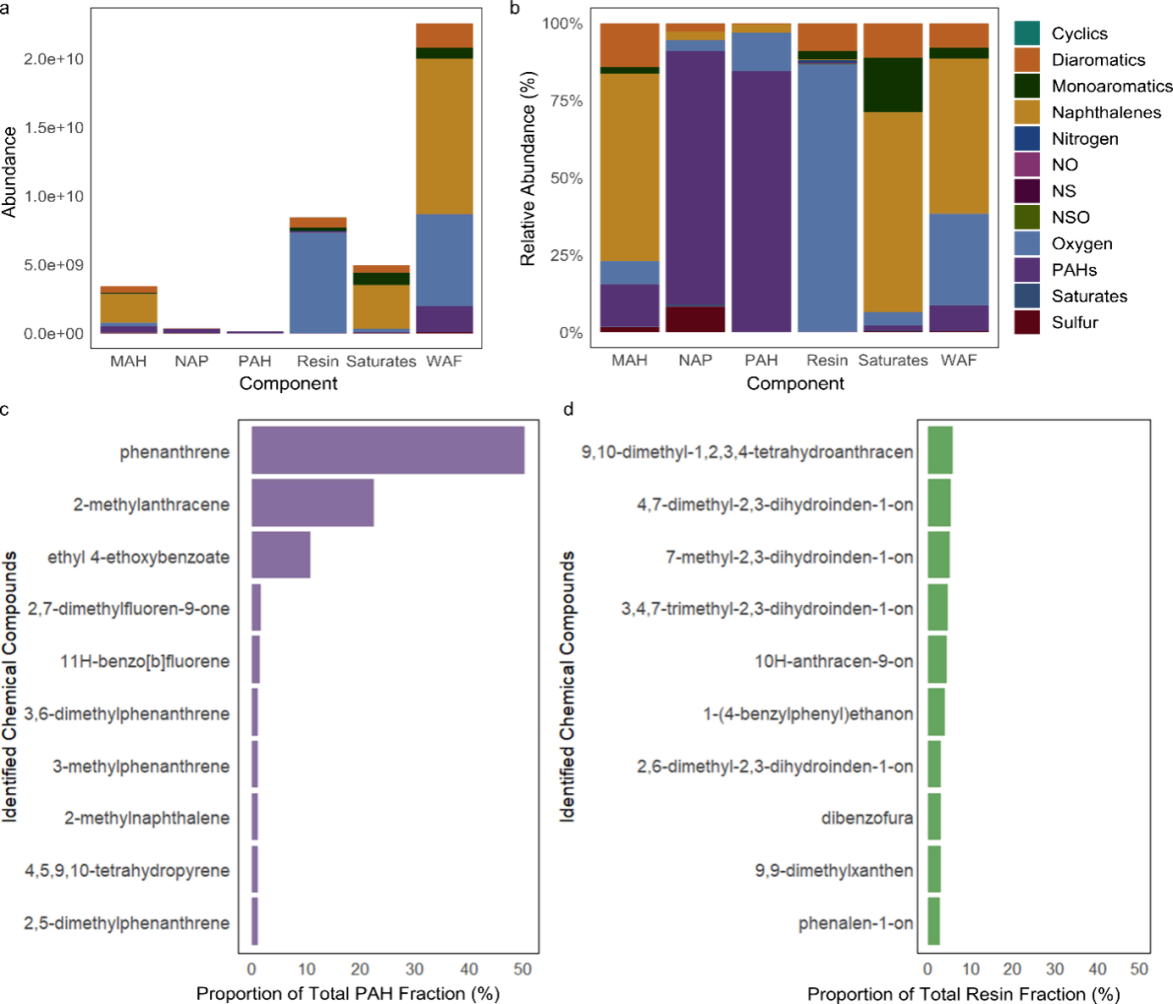
Chemical composition of WAF and its fractions. (a) Stacked bar chart of cumulative chemical class abundance across WAF fractions. (b) Relative abundance of chemical classes per fraction, illustrating compositional variability. (c, d) Relative abundance of the 10 most common compounds in (c) PAH and (d) Resin fractions.

The Resin fraction consisted exclusively of oxygenated and heteroatom-containing species. High-molecular-weight PAHs were concentrated within the PAH fraction. Naphthalenes were most enriched in the MAH and Saturate fractions, likely due to column overloading during fractionation, reflecting their high relative abundance in WAF (Figure 3a,b).

The PAH fraction was comprised of three dominant peaks, assigned as phenanthrene (50% abundance), 2-methylanthracene (15%), and ethyl 4-ethoxybenzoate (11%) (Figure 3c). The latter is technically a monoaromatic ester but coeluted in the PAH fraction due to its retention properties. Collectively, these compounds accounted for 76% of the relative abundance (Figure 3c). The remaining identified peaks in the PAH fraction were detected at lower relative abundances. By contrast, the Resin fraction was highly heterogeneous, with over 190 identified constituents. No single peak exceeded 5% abundance, underscoring its chemical complexity. The 10 most abundant constituents are shown in Figure 3d. Compositional profiles for MAH, Saturate, and NAP fractions were also determined (Figure S4), but were not prioritized for further behavioral analysis due to their more limited contribution to the WAF neurobehavioral signature.

### 3.4 Association of Total WAF, WAF Fraction, and Chemical Component Behavior Patterns

To assess whether behavior effects observed in exposed larvae were driven by specific chemicals or multiple constituents, we tested representative compounds from the PAH (phenanthrene, 2-methylanthracene, ethyl 4-ethoxybenzoate) and Resin (7-methyl-1-indanone, 1H-phenalen-1-one, dihydrocarvone) fractions (Tables S2 and S3). Among PAHs, exposure to phenanthrene (50% of the PAH fraction) triggered pronounced behavior effects (Figure 4b,c and Figure S5a), including habituation impairment observed in larvae exposed to the PAH fraction (Figure S2c). In contrast, exposure to 2-methylanthracene or ethyl 4-ethoxybenzoate resulted in alterations in dark-phase end points (BSL4, VMR5) (Figure 4a,c and Figure S5b,c) but failed to reproduce the hyperactivity elicited by exposure to the PAH fraction (Figure S5a–c).

**Figure 4:**
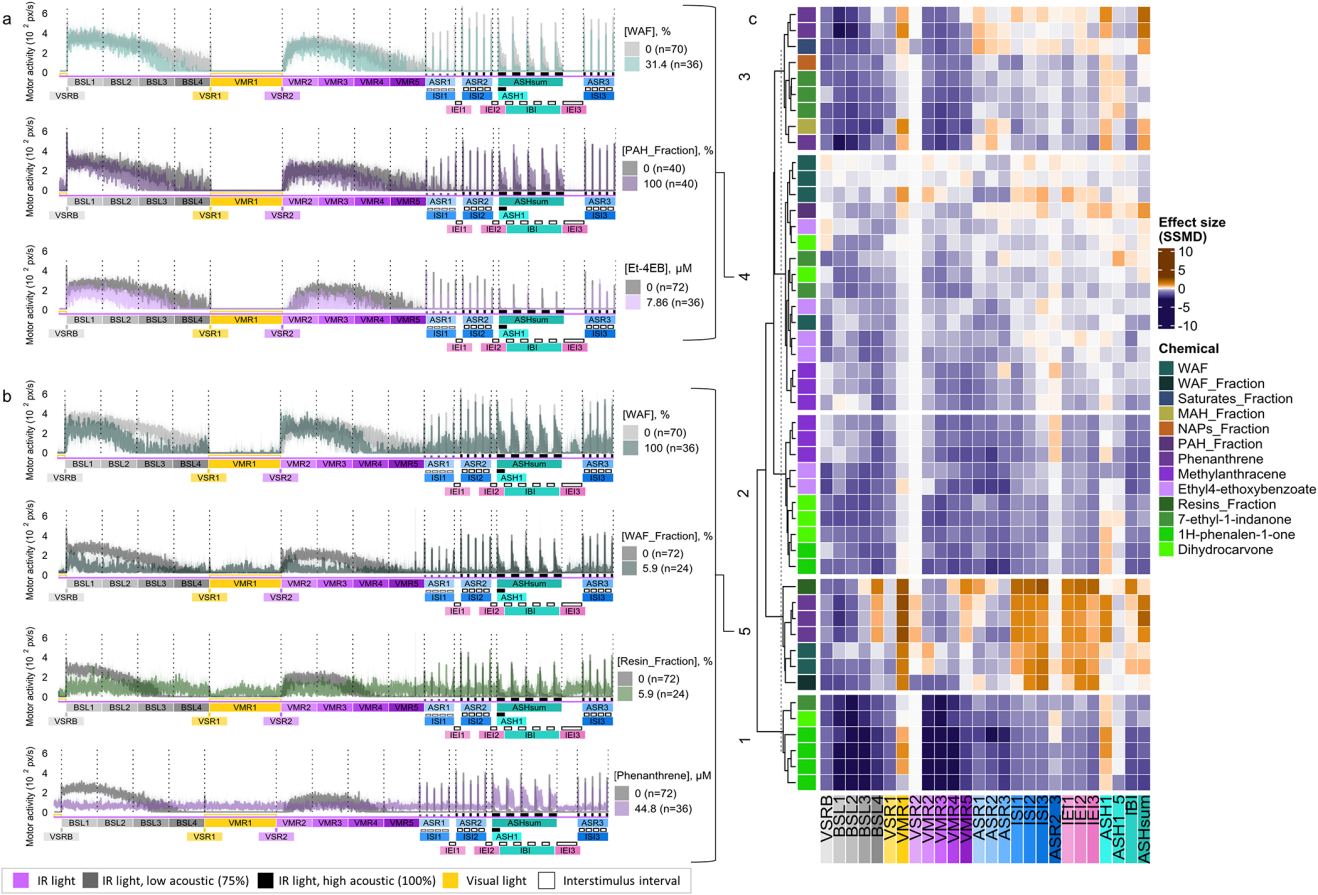
Behavior signatures induced by exposure to WAF, fractions, and individual chemical constituents. (a) Representative profiles from cluster 4. (b) Representative activity of zebrafish in cluster 5. Median ± 95% confidence internal (CI) shown; controls in gray (*n* = 70–72). (c) Hierarchical clustering of 26-end point profiles from larvae exposed to the WAF, WAF fractions, or representative PAH (purple shades or Resin (green shades) chemicals. Control groups: HBSS for WAF, blank fractions for subcomponents, and 0.4% DMSO for chemicals. Heatmap shows SSMD effect sizes (orange = hyperactivity, blue/purple = hypoactivity; *n* = 33–44). WAF/fraction data reused from Figures 1 and 2. Et-4EB, Ethyl-4-ethoxybenzoate.

Hierarchical clustering was used to integrate data from the WAF concentration–response series (Figure 1), WAF fractions (Figure 2), and representative individual compounds from the PAH and Resin fractions (Figure 4). Cluster 1 comprised several concentrations of Resin fraction components 1H-phenalen-1-one, 7-ethyl-1-indanone, and dihydrocarvone, characterized by general hypoactivity, intermittent light-phase hyperactivity, and habituation impairment (Figure 4c and Figure S6). Cluster 2 exhibited hypoactivity across all end points and included concentrations of 2-methylanthracene, ethyl 4-ethoxybenzoate, 7-ethyl-1-indanone, and dihydrocarvone (Figure 4c and Figure S6). Cluster 3, comprised phenanthrene, 7-ethyl-1-indanone, and contributions from the Saturate, NAP, and MAH fractions (Figure 4c and Figure S6) was characterized by dark-phase hypoactivity, light-phase hyperactivity, and altered habituation. Cluster 4 included concentrations of the total WAF, the PAH fraction, and several concentrations of ethyl 4-ethoxybenzoate and 2-methylanthracene (PAH constituents), as well as dihydrocarvone and 1H-phenalen-1-one (Resin constituents). Behavioral profiles in this cluster were defined by hypoactivity during dark phases and acoustic stimulation (Figure 4a,c). In contrast, Cluster 5 included the full Resin fraction, WAF fraction, higher WAF concentrations, and multiple phenanthrene concentrations. This cluster was distinguished by pronounced hyperactivity during light-phase stimulation (VMR1) and interstimulus intervals (ISI, IEI) (Figure 4b,c).

### 3.5 Recapitulation of WAF and WAF Fraction Effects Using a Chemically Defined Artificial Mixture

To determine whether the behavioral phenotype elicited by exposure to the PAH fraction could be reproduced by its primary chemical constituents, we tested a defined mixture composed of phenanthrene, 2-methylanthracene, and ethyl 4-ethoxybenzoate formulated based on the relative abundances of the three major components (Figure 5a,b). In the VAMR assay, larvae exposed to the mixture at ≥4.4 μM displayed significant habituation deficits, phenocopying PAH fraction effects on ASH1 and ASHsum end points (Figure 5c and Figures S2c and S7). This phenotype was primarily driven by phenanthrene exposure (Figure 4b). At higher concentrations (25.1–80 μM), the mixture also induced light-phase hyperactivity (VMR1) and increased activity during interstimulus intervals (ISI1–3, IEI1–3, IBI), which were not observed following native PAH fraction exposure (Figure 5c,d and Figure S2c). At lower concentrations (0.2–0.4 μM), the artificial mixture reproduced the PAH fraction’s dark-phase behavioral profile, including hypoactivity effects (BSL4, VMR5) (Figure 5c,d).

**Figure 5:**
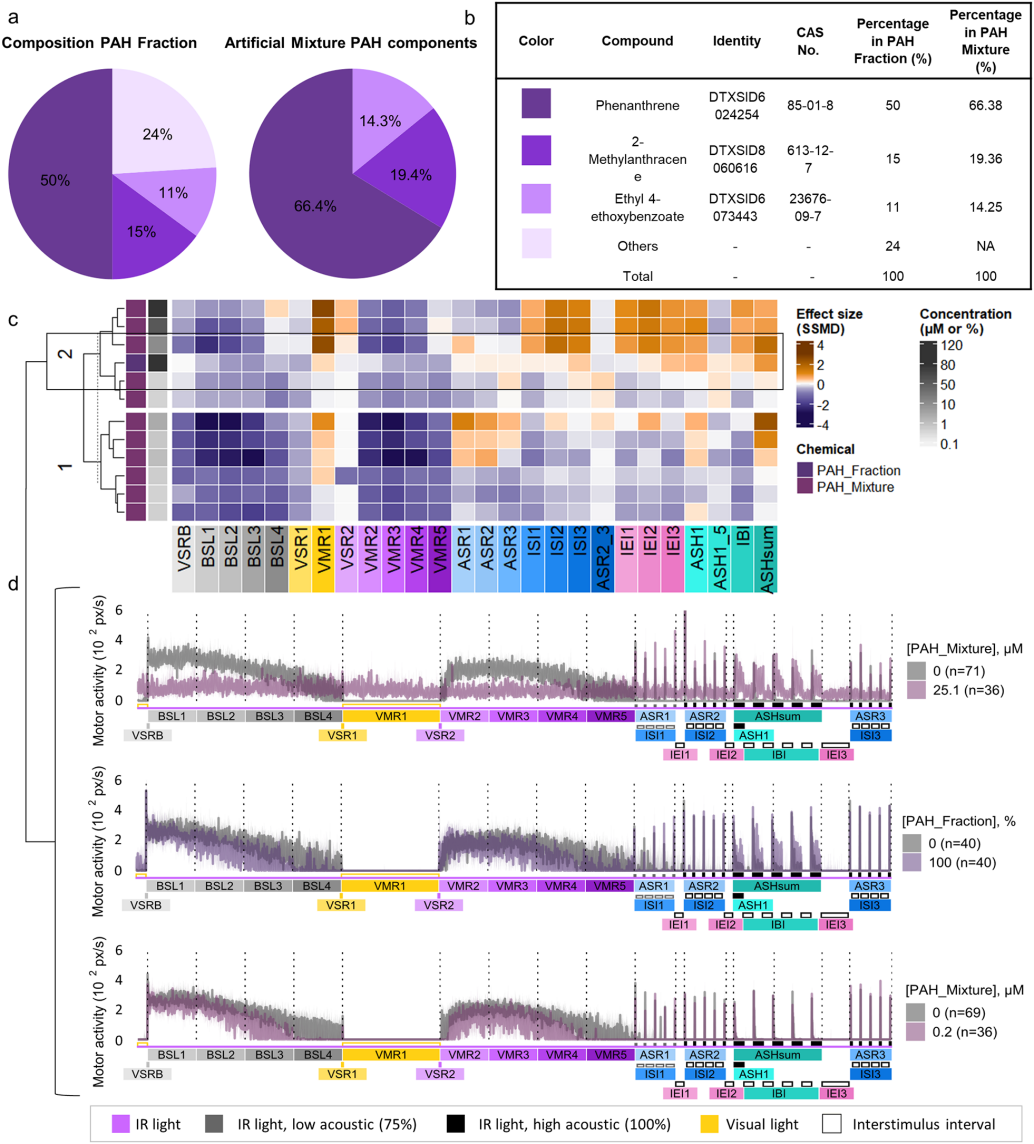
Exposure to artificial mixture partially recapitulates PAH fraction behavior effects. (a) Pie charts showing the relative chemical composition of the PAH fraction (left) and the defined artificial mixture (right). (b) Composition table showing the identity, CAS number, and proportional contribution of each constituent; colors correspond to the pie-chart segments in panel (a). (c) Hierarchical clustering of 26-end point behavioral profiles for PAH fraction (100%) and artificial mixture (0.2–80 μM). Effect size heatmaps: orange = hyperactivity, blue = hypoactivity (*n* = 36–40 per group). PAH fraction vs blank; artificial mixture vs 0.4% DMSO control. (d) Median motor activity of larvae exposed to PAH fraction (100%) and artificial mixture (0.2, 25.1 μM). Shaded areas show 95% CI; group-specific controls in gray (*n* = 40–71). PAH fraction data reused from Figure 2.

### 3.6 Identifying Putative Mode-of-Action Information for WAF and Fraction-Dependent Behavior Effects

While this work identified key chemical contributors to WAF fraction-induced behavior effects, the potential underlying mechanism(s) of action (MoAs) remain unclear. To generate hypotheses regarding potential neuroactive pathways disrupted in WAF and fraction-exposed cohorts, we applied the VAMR NAM fingerprinting system, which systematically compares behavioral phenotypes to a database of 63 reference chemicals with known MoAs targeting key pathways essential for neurodevelopment or neurotransmission or toxicologically relevant signaling pathways.^21^ Integrating phenotypic data from WAF and fraction exposures into this framework enabled identification of candidate molecular targets and pathways potentially mediating observed neuroactivity. Hierarchical clustering of WAF and fractions fingerprints revealed diverse neuroactive signatures (Figure 6a and Figure S8). The Resin fraction clustered near bicuculline, a γ-aminobutyric acid A receptor (GABA_A_R) antagonist. The PAH fraction and lower WAF concentrations grouped within Cluster 13, a chemically heterogeneous cluster without a single dominant MoA. Notably, the PAH fraction formed a subcluster with a specific concentration of ODQ, a nitric oxide-cGMP pathway modulator, which also appeared in Cluster 15 near the 75% and 100% WAF exposure. Distinct groupings were observed for Saturate and WAF fractions, which coclustered with etomidate, a GABA_A_R agonist and butoxyethanol, a predicted nonspecific toxicant.^35^ MAH and NAP fractions aligned with four reference compounds spanning multiple MoAs (Table S4), including GW7647 a Peroxisome Proliferator-Activated Receptor Alfa (PPARα) agonist, a microbial enzyme inhibitor, a dopamine receptor antagonist, and a ryanodine receptor (RyR) inhibitor. At higher concentrations, exposure to total WAF (56.6%, 75%, 100%) grouped within Cluster 15, enriched with compounds known to interfere with neurodevelopmental signaling. This cluster included modulators of mechanistic target of rapamycin (mTOR) (MHY1485), retinoid X receptor (RXR) (HX-531), and bone morphogenetic protein 4 (BMP4) (SB4), as well as both agonists and antagonists of Peroxisome Proliferator-Activated Receptor Delta (PPARδ). These associations support the hypothesis that WAF-mediated neurotoxicity involves receptor-level disruption of key developmental regulatory pathways (Figure 6a,b).

**Figure 6:**
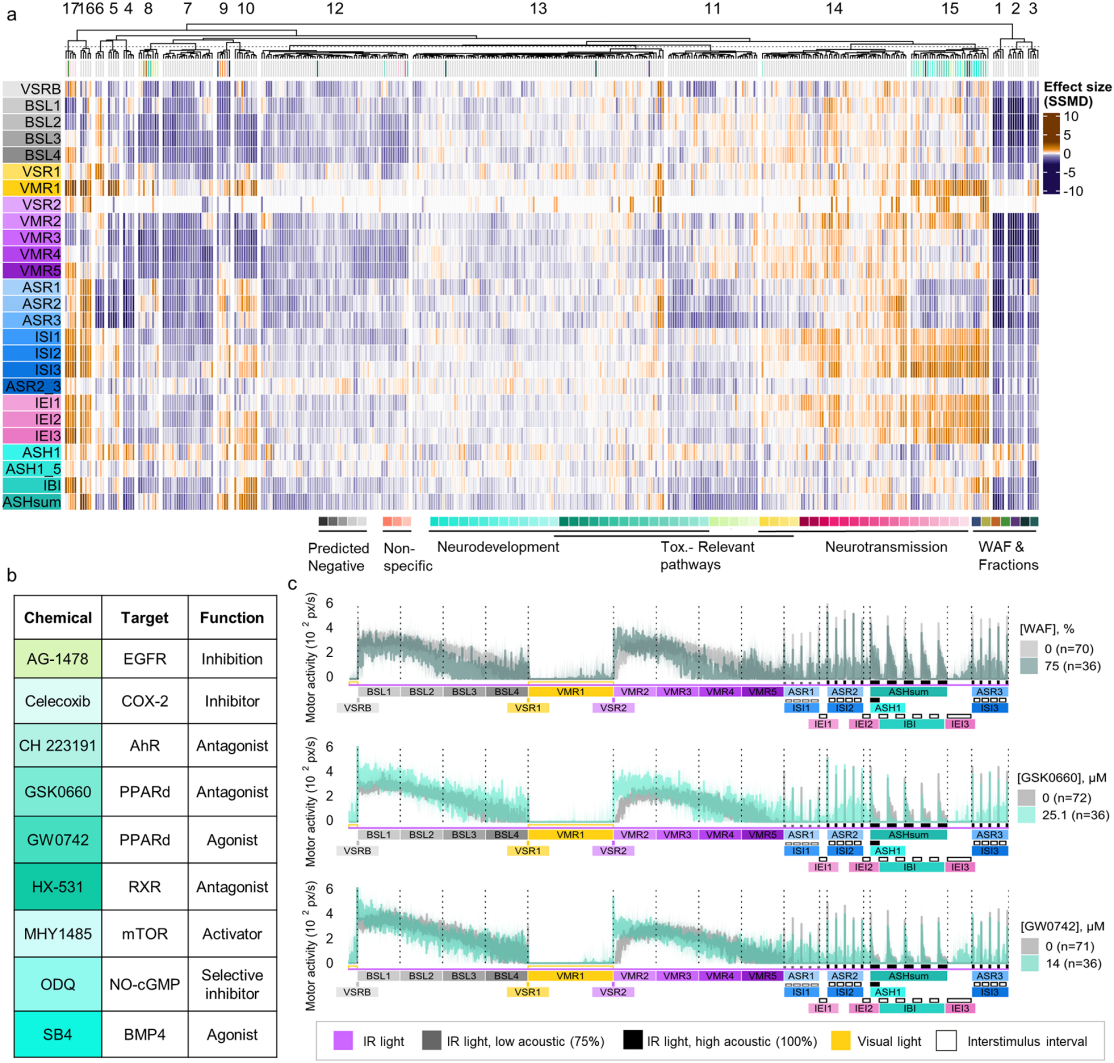
Neuroactive profiling suggests mechanistic overlap between WAF, its fractions, and neuroactive reference chemicals. (a) Hierarchical clustering of 63 reference compounds, WAF, and WAF fractions based on SSMD-derived behavioral profiles across the 26-end point VAMR assay. Shades of orange indicate increased activity while shades of blue/purple indicate reduced swimming activity (*n* = 36–40 larvae/group). (b) Targets and biological functions of Cluster 15 reference compounds. (c) Median motor activity profiles for zebrafish exposed to WAF (75%), GSK0660 (25.1 μM, PPARδ antagonist), and GW0742 (14 μM, PPARδ agonist). Shaded areas represent 95% confidence intervals; controls are shown in gray (*n* = 70–72). GSK0660 and GW0742 profiles from ref 21.

## 4 Discussion

Exposure to crude oil has been shown to alter swimming behavior in simple, zebrafish-based light-dark transition tests,^14,36–38^ yet the mechanisms driving these effects remain poorly understood. To address this gap, we used a phenotypically rich neurobehavioral assay, comprised of 26 end points including learning and memory-related end points,^21,39^ integrating behavioral profiling with chemical fractionation to resolve the contribution of WAF fractions and constituents to observed behavior effects.

Acute exposure to WAF elicited concentration-dependent disruptions in stimulus-evoked behaviors, including hyperactivity during the light period, the first phase of the dark period, and the interstimulus intervals, reduced swimming activity during the final phases of the dark period, and reduced acoustic startle responses. While earlier studies reporting reduced swimming activity in simple light-dark transition assays,^36,37^ the present study revealed a range of novel increased and decreased swimming behaviors across the phenotypically rich VAMR assay. This difference from historic data is likely due to the use of an acute, 40 min exposure period. We opted for a shorter exposure period based on earlier work showing that fingerprints derived from acute exposures tend to be more unique, and therefore more applicable for mode-of-action prediction.^21,22,40–43^

To better understand potential drivers of neuroactivity effects, WAF fractionation was used to enable mechanistic dissection of constituent-specific neuroactive signatures. Exposure to all fractions replicated the dark-phase hypoactivity observed in larvae exposed to the unfractionated, total WAF, suggesting that multiple chemical classes contribute to this phenotype. Similar reductions in swimming activity have also been reported in other species, such as marine copepods exposed to crude oil and chemically dispersed oil.^44^ Beyond locomotor changes, a key finding was that exposure to PAH, MAH, or NAP fractions impaired habituation learning, a fundamental nonassociative learning process marked by reduced responsiveness to repeated stimuli.^45^ Habituation is highly conserved across taxa and reflects core aspects of sensory filtering, attention, and neural adaptation.^45,46^ Impaired habituation was observed in larvae exposed to these fractions, consistent with disrupted experience-dependent plasticity.^47^ While impaired learning has not been previously assessed in larval zebrafish studies focused on oil-related toxicity, developmental exposure of zebrafish embryos to benzo­[a]­pyrene, a PAH WAF constituent, was reported to impair associative learning and memory formation in adulthood, as demonstrated by reduced performance in an active avoidance shuttlebox test.^48^ These data support the concept that exposure to PAHs potentially disrupts learning in vertebrate organisms. Notably, WAF exposure did not directly impair habituation learning. However, in the ASHsum end point, which reflects the cumulative startle response during habituation training, exposure to the highest WAF concentration (100%) increased activity relative to the control. The lack of habituation-related effects in WAF-exposed larvae, despite strong effects observed following exposure to individual fractions, suggests masking or attenuation of habituation effects within the full mixture. This may reflect receptor-level antagonism, compensation, or nonadditive effects, underscoring the need for component-resolved mixture assessment.^49,50^ We also note that adsorption of hydrophobic WAF constituents to plastic exposure wells may have reduced effective aqueous concentrations; however, because all groups were treated identically, relative comparisons across treatments remain valid. Hydrophobic chemicals such as PAHs are also prone to sorption to glass surfaces, indicating that this behavior is a general property of these compounds rather than a material-specific artifact.^51^

Exposure to PAH or Resin fractions produced behavior profiles that were the most similar to WAF-induced effects, reflecting their dominant contribution to the mixture phenotype. While PAH toxicology is well characterized,^48,52–54^ the neuroactivity potential of resin-type compounds remains underexplored. In this study, exposure to the Resin fraction distinctly caused persistent hyperactivity across most assay phases, including typically quiescent intervals. Individual exposure to the three most prevalent compounds detected in the Resin fraction failed to recapitulate the pronounced hyperactivity observed following exposure to the Resin fraction. This discrepancy likely reflects both analytical and toxicological factors. From an analytical perspective, the applied fractionation procedure involved a single elution step, which may not have fully recovered more polar or heteroatom-rich resin components. Previous studies have shown that petroleum resins contain subfractions requiring stronger eluents (e.g., ethyl acetate in modified SARA protocols) to isolate oxygenated or sulfur-containing compounds.^55,56^ Moreover, comprehensive characterization of crude oil- and produced-water-derived mixtures has demonstrated that detection of these polar constituents often requires complementary ionization modes and chromatographic methods,^57^ which were beyond the analytical scope of the current study. From a toxicological standpoint, exposure to the Resin fraction produced a unique and widespread hyperactivity profile not recapitulated by individual compounds, suggesting the presence of undetected trace-level substances within the Resin fraction that may act individually or synergistically to drive the observed behavioral effects. Future work should evaluate the neuroactive potential of polar or heteroatom-rich Resin components. Another explanation for the disparate results observed following exposure to the Resin fraction or individual fraction components may relate to loss of polar constituents during solvent exchange and DMSO reconstitution. Resin subclasses are prone to adsorption and require stronger eluents for complete recovery, as demonstrated in modified SARA fractionation.^56^ Because resin definitions are method-dependent, with polar and heteroatom-rich species often underrepresented,^58^ selective losses could have reduced the presence of potent neuroactivity drivers in our reconstituted samples.

In contrast, the PAH fraction was less complex, with phenanthrene comprising ∼50% of the total peak area. Behavior testing of the three most abundant PAH constituents, both individually and as a representative mixture partially reproduced PAH fraction-associated phenotypes, including habituation deficits and phase-specific hypoactivity. Similar findings were reported for zebrafish exposed to 10 PAHs and three defined mixtures,^53^ where mixture effects reflected the activity of the most potent constituents. Although phenanthrene was not a key behavioral driver in that study,^53^ here we found that exposure altered both light- and dark-phase activity and strongly impaired habituation learning. The broader behavioral profile observed here was again likely due to the use of a more phenotypically complex behavior assays with multiple stimuli and capturing a range of end points.^21,22^ While the three-component artificial mixture captured key aspects of the PAH fraction phenotype, its inability to fully phenocopy the original fraction at a single concentration highlights the emergent properties of complex chemical interactions. At low concentrations (0.2–0.4 μM), exposure to the artificial mixture reproduced the PAH fraction’s dark-phase hypoactivity, but at higher concentrations (25.1–80 μM) it induced hyperactivity across normally quiescent periods (e.g., during the light period and interstimulus intervals). In contrast, weak hyperactivity in these end points was observed in larvae exposed to the native PAH fraction. This divergence may reflect masking effects or receptor antagonism from other PAH fraction constituents that were not included in the artificial mixture. While the three components used to simulate the PAH fraction represented ∼76% of the fraction, even low-abundance or individually “inactive” chemicals have been shown to cause unexpected toxicity in mixtures, as observed previously with a PAH submixture.^53^ Moreover, crude oil-derived mixtures are often enriched in alkylated PAHs that differ in uptake and potency from parent compounds.^3^ Thus, while the artificial PAH mixture recapitulated some key neuroactivity effects, additional constituents could explain the observed divergence in neuroactivity patterns. Future work should therefore include more constituents to more accurately simulate the PAH fraction.

To predict potential mechanisms by which exposure to WAF or WAF fractions alter neuroactivity in zebrafish, we applied a neurobehavioral fingerprinting approach, comparing VAMR-derived profiles to a curated reference library of compounds with known neuroactive targets.^21^ Exposure to the WAF also uniquely clustered with profiles derived from exposure to both a PPARδ agonist (GW0742) and antagonist (GSK0660). This supports the concept that ppar-dependent signaling could be a target of WAF-related chemical constituents. Notably, exposure to WAF or the PPARδ modulators induced persistent hyperactivity in light-phase transitions, especially during the initial part of the dark period (i.e., VMR2). Previous work demonstrated that persistent visual startle response hyperactivity observed in PFOS-exposed zebrafish was causally mediated by PPARδ signaling.^59^ Rodent^60^ and human^61^ studies have similarly linked PPARδ signaling to locomotor regulation, supporting its conserved role in modulating behavior across vertebrates.^60^ WAF fractions did not cluster with PPARδ modulators, potentially representing novel chemistries present in the WAF that were not reflected in any single WAF fraction.

Comparison of behavioral profiles elicited by exposure to the WAF and its individual fractions revealed distinct clustering patterns. For example, behavior profiles elicited by exposure to Resin or PAH fractions clustered closely with Bicuculline, a reference compound targeting GABAergic signaling. The GABA_A_R is a key regulator of inhibitory neurotransmission and synaptic plasticity^62,63^ and is highly conserved across vertebrates, including zebrafish, mice, and humans.^64^ GABA_A_R antagonists, such as bicuculline or picrotoxin, increase locomotor responses during acoustic startle and typically quiescent end points (e.g., interstimulus intervals, interend points intervals and light phase), whereas agonists such as etomidate or propofol suppress activity and blunt stimulus-evoked responses.^21,65–68^ Previous zebrafish studies reported that GABA_A_R antagonists induce hyperactivity and seizure-like behaviors by disrupting inhibitory signaling in the optic tectum, leading to altered visual processing and increased neuronal excitability.^68^ Impaired habituation, observed in PAH exposed larvae, has also been linked to GABA_A_R dysfunction characterized by α5-subunit–mediated tonic inhibition.^69–75^ Beyond WAF-related compounds, disruption of GABAergic signaling has been reported following exposures of structurally diverse environmental contaminants, including chlorophene,^22^ PFOS,^76^ TBBPA,^21^ fipronil,^77^ linuron,^78^ and thiacolprid,^79^ highlighting the sensitivity of this pathway to chemical interference. Together, these findings suggest that disruption of GABA_A_R signaling may represent a shared MoA driving neuroactivity effects observed in larvae exposed to the WAF, PAH, or Resin fractions. In addition to potential disruption of GABAergic signaling, the MAH and PAH fractions clustered with a reference compound targeting dopamine receptors and MAH also aligned with a ryanodine receptor (RyR) modulator. Interestingly, these WAF fractions and receptor modulators alter habituation learning (Current study and ref 21). These discordant associations may reflect masking or antagonistic effects in the more complex WAF mixture relative to the fractions. Although behavioral fingerprinting supports hypothesis generation regarding putative targets such as GABA_A_ receptors or PPARδ, these predictions require experimental evaluation. Future work should employ mechanistic strategies such as pharmacological manipulation or genetic engineering to evaluate these predictions.

In summary, our findings demonstrate that crude oil WAF-induced neurobehavioral disruption likely arises from the combined action of PAHs, oxygenated and polar constituents, stemming from interference with multiple receptor-mediated signaling pathways. Multiend point profiling across 26 behavior-based measures of visual-motor function, startle responses, and habituation learning enabled sensitive evaluation of neuroactive effects. By coupling phenotypically rich behavior fingerprints with chemical fractionation and mode-of-action fingerprinting, this study provides mechanistic insight into the specific components and pathways that potentially drive crude oil-induced disruption of neurobehavioral function in early life-stage fish. These findings have ecological implications, as neurodevelopmental disruption may impair critical behaviors necessary for survival that could potentially affect population resilience.^80–82^

## Supplementary Material



## Notes

Zebrafish husbandry details; behavioral testing protocol; (Figures S1–S8) behavioral response profiles, clustering results, chemical composition of WAF, WAF fractions, and individual constituents; and (Tables S1–S4) GC×GC-MS compound annotations, identified PAH and Resin constituents, and toxicity summary of top resin fraction compounds and reference chemical clusters (PDF)

## Abbreviations

ASH: acoustic startle habituation
ASHsum: cumulative motor activity across all five habituation bouts
ASR: acoustic startle response
BMP4: bone morphogenetic protein 4
BSL: baseline motor activity
CI: confidence internal
COX-2: cyclooxygenase-2
CROSERF: Chemical Response to Oil Spills: Ecological Research Forum
DCM: dichloromethane
DMSO: dimethyl sulfoxide
EDA: effect-directed analysis
EI: electron ionization
GABA_A_R: gamma-aminobutyric acid type A receptor
GC×GC-MS: comprehensive two-dimensional gas chromatography–mass spectrometry
HBSS: Hanks’ balanced salt solution
HPLC: high-performance liquid chromatography
IBI: interbout interval
IEI: interend point interval
ISI: interstimulus interval
MAH: monoaromatic hydrocarbon
MoA-s: mode of action
mTOR: mechanistic target of rapamycin
*m*/*z*: mass-to-charge ratio
NAM: new approach methodology
NAP: naphthalene
OBI: outbred laboratory zebrafish line
PAH: polycyclic aromatic hydrocarbon
PARC: European Partnership for the Assessment of Risks from Chemicals
PLS: partial least-squares
PPAR: peroxisome proliferator-activated receptor
PPARα: peroxisome proliferator-activated receptor alfa
PPARδ: peroxisome proliferator-activated receptor delta
RIs: retention indices
RT: retention times
RXR: retinoid X receptor
RyR: ryanodine receptor
SSMD: strictly standardized median difference
TIC: total ion chromatogram
UFZ: Helmholtz Centre for Environmental Research
VAMR: visual and acoustic motor response
VMR: visual motor response
VSR: visual startle response
VSRB: visual startle response baseline
WAF: water-accommodated fraction
