# Supplementary material for: Identification of Neuroactive Chemicals in Crude Oil-Derived Water-Accommodated Fractions
Source: Environ Sci Technol. 2025 Dec 22;60(1):181–95. doi: 10.1021/acs.est.5c06859 (PMC12810237; doi:10.1021/acs.est.5c06859)
Supplement: Supplementary file 1 [file es5c06859_si_001.pdf]

# Supporting Information for Identification of Neuroactive Chemicals in Crude Oil-Derived Water Accommodated Fractions

*Nadia K. Herold[1], Lisbet Sørensen[2,3], Mari E. Creese[2], Jasmine Nahrgang[4], Nicole Schweiger[1], Tamara Tal[1,5]*.*

[1]Ecotoxicology Department, Chemicals in the Environment Research Section, Helmholtz Centre for Environmental Research – UFZ, 04318 Leipzig, Germany
[2]SINTEF Ocean, Climate and Environment, 7465 Trondheim, Norway

[3] Department of Chemistry, Norwegian University of Science and Technology (NTNU), 7491 Trondheim, Norway

[4] Department of Arctic and Marine Biology, UiT The Arctic University of Norway, 9037 Tromsø, Norway

[5]Medical Faculty, Leipzig University, 04103 Leipzig, Germany

*Corresponding author: tamara.tal@ufz.de

Number of pages: 33
Number of figures: 9
Number of tables: 4

---

**Contents**:

•Section S1 – Zebrafish Husbandry (full version) ……………………………………………S2

•Section S2 – Behavioral Protocol Details……………………………………………………S3

•Table S1 – Overview of compounds used for training and testing the GC×GC retention models ………………………………………………………………………………………..S4

•Figure S1 – Motor activity profiles and behavioral endpoint responses of zebrafish exposed to WAF……………………………....…………………………………………….……..S7

•Figure S2 – Behavioral responses to WAF fractions…………………………………….……S9

•Figure S3 – Clustering and multidimensional scaling of behavioral profiles for WAF and fractions……………………………………………………….…………………....S15

•Figure S4 – GC×GC-MS chemical composition of WAF fractions (MAH, Saturates, NAPs)………………………………………………………………….…S16

•Table S2 – Identified and tested individual chemicals for PAH and Resin Fractions ..….S17

•Table S3 – Toxicity Summary of the Top 10 Resin Fraction Compounds ………….…..S18

•Figure S5 – Behavioral data for individual PAH constituents ..………………….…..……...S19

•Figure S6 – Behavioral data for individual Resin constituents……………………………...S25

•Figure S7 – Behavioral responses to artificial PAH mixture …………………………......S31
.
•Figure S8 – Comparative clustering of WAF/fraction profiles against fingerprinting library of 63 reference chemicals ………..……………………..……………………...…S33

•Table S4 – Overview of reference chemical clusters associated with neuroactive fingerprinting hits for WAF and isolated fractions…………………………………….…..…S34

**Section S1 | Zebrafish Husbandry (full version)**

2.1 Zebrafish husbandry

All procedures involving the care and handling of zebrafish (Danio rerio) were conducted in compliance with established guidelines and regulations. Approval was granted by the local government authority (Landesdirektion Sachsen, Geschäftszeichen DD24-5131/252/7), ensuring adherence to ethical and legal standards. Zebrafish, resulting from an outcross between WIK and the in-house 'UFZ-OBI' strain, were housed in 26 L glass tanks at a density of approximately three fish per liter. Water quality parameters were routinely monitored to ensure optimal conditions. pH levels were maintained between 7 and 8, water hardness ranged from 2 to 3 mmol/L, and nitrate concentrations remained below 2.5 mg/L. Nitrite and ammonia levels were controlled at <0.025 mg/L and <0.6 mg/L, respectively, while oxygen saturation was kept within 87–91 %. Adult zebrafish received dry food (Sparos) twice daily and shell-free artemia (Sanders) once daily from Monday to Friday, with both food sources provided once on weekends. Zebrafish were maintained on a 14-h light, 10-h dark cycle at 28 °C and were bred every 1-2 weeks by placing trays in on-rack glass tanks. The following morning, embryos at the zygote stage were collected and selected based on the criteria outlined by Kimmel et al. (1995) using a dissection microscope (Olympus SZx7-ILLT).

## Section S2 | Behavioral Protocol Details

### 2.7 Automated behavior testing

The behavior assay conducted in this study was adapted from previous research32,24. Sixty min before acute chemical exposure, zebrafish larvae in 96-square well microtiter plates were exposed to light. After an additional 40 min, plates were transferred to the Zebrabox behavior device (ViewPoint), where they remained under continuous infrared illumination. Video recordings were captured at 25 frames per second using ZebraLab video tracking software (ViewPoint) set to 'quantization mode' with a sensitivity threshold of 15, enabling measurement of locomotor activity based on changes in pixel intensity per second.

The behavior test contained two modalities including visual and acoustic stimuli. Visual stimuli consisted of light conditions at 0 lux (dark) and 13.5 klux (light), with light intensity measured at using an LI-250 light meter (LI-COR). Acoustic stimuli were carried at 300 Hz, with sound intensities of 65 dB for the first acoustic startle response (ASR1) and 75 dB for all following responses (ASR2, ASR3, ASH1-5). The experiment began with a 21 min acclimation period at 28 °C, during which larvae were first exposed to 1 min of light (13.5 klux), followed by 20 min of dark (0 klux). The visual startle response at baseline (VSRB), encompassing the transition from light to dark conditions, triggered a brief motor response lasting approximately 1 s. The light was then turned on for 10 min (VMR1) before turning off for the rest of the test. The first 20 min of dark comprised the VMR2-5 endpoints after which, the acoustic stimuli phase began. First, five lower-volume (65 dB) acoustic stimuli were delivered, each spaced 1 min apart (ASR1). Following a 1 min inter-endpoint interval (IEI1) between the ASR1 and ASR2 endpoints, a second acoustic phase began with five higher-volume (75 dB) acoustic stimuli, also spaced 1 min apart (ASR2). The 1 min gaps between stimuli within each stimulus were used to define inter-stimulus intervals (ISI1-3). After ASR2, a second inter-endpoint interval (IEI2) preceded the habituation phase. This phase consisted of five bouts (ASH1–5), each composed of 30 high-intensity acoustic stimuli (75 dB) delivered at 1 s intervals. The 1 min periods between the five habituation bouts were defined as the inter-bout interval (IBI). Following the final bout (ASH5), a third inter-endpoint interval (IEI3) was introduced before the onset of the final acoustic phase (ASR3), which again included five high-volume acoustic stimuli (75 dB) spaced 1 min apart and followed the same structure as ASR2.

**Table S1 |** Overview of compounds used for training and testing the GC×GC retention models

| Compound class | Compound Name | CAS | Rt 1D (min) | Rt 2D (s) |
|---|---|---|---|---|
| n-alkanes | nC12 | 112-40-3 | 12.95 | 0.53 |
| n-alkanes | nC13 | 629-50-5 | 15.61 | 0.54 |
| n-alkanes | nC14 | 629-59-4 | 18.20 | 0.59 |
| n-alkanes | nC15 | 629-62-9 | 20.66 | 0.59 |
| n-alkanes | nC16 | 544-76-3 | 22.99 | 0.59 |
| n-alkanes | nC17 | 629-78-7 | 25.21 | 0.64 |
| n-alkanes | nC18 | 593-45-3 | 27.33 | 0.65 |
| n-alkanes | nC19 | 629-92-5 | 29.36 | 0.65 |
| n-alkanes | nC20 | 112-95-8 | 31.26 | 0.69 |
| n-alkanes | nC21 | 629-94-7 | 33.12 | 0.70 |
| n-alkanes | nC22 | 629-97-0 | 34.88 | 0.69 |
| n-alkanes | nC23 | 638-67-5 | 36.58 | 0.71 |
| n-alkanes | nC24 | 646-31-1 | 38.21 | 0.76 |
| n-alkanes | nC25 | 629-99-2 | 39.71 | 0.77 |
| n-alkanes | nC26 | 630-01-3 | 41.25 | 0.79 |
| n-alkanes | nC27 | 593-49-7 | 42.72 | 0.82 |
| n-alkanes | nC28 | 630-02-4 | 44.11 | 0.83 |
| n-alkanes | nC30 | 638-68-6 | 46.79 | 0.89 |
| n-alkanes | nC32 | 544-85-4 | 49.31 | 1.01 |
| n-alkanes | nC34 | 14167-59-0 | 53.82 | 1.88 |
| n-alkanes | nC35 | 630-07-9 | 55.91 | 2.33 |
| NSO | Benzo[h]quinoline | 230-27-3 | 26.00 | 3.60 |
| NSO | Xanthone | 90-47-1 | 27.37 | 3.62 |
| OCPs | Tripropyl phosphate | 513-08-6 | 16.90 | 1.78 |
| OCPs | Tributyl phosphate | 126-73-8 | 23.31 | 1.59 |
| OCPs | Tris-(2-chloroethyl)phospate | 115-96-8 | 25.05 | 3.57 |
| OCPs | Triphenyl phosphate | 115-86-6 | 36.98 | 4.06 |
| OCPs | Tris(2-ethylhexyl)phosphate | 78-42-2 | 38.94 | 1.13 |
| PAH | Naphthalene | 91-20-3 | 11.80 | 2.09 |
| PAH | 1-Methylnaphthalene | 90-12-0 | 15.12 | 2.21 |
| PAH | Biphenyl | 92-52-4 | 16.83 | 2.25 |
| PAH | 2,3-Dimethylnaphthalene | 581-40-8 | 17.59 | 1.99 |
| PAH | 2,6-Dimethylnaphthalene | 581-42-0 | 18.39 | 2.21 |
| PAH | Acenaphthylene | 208-96-8 | 18.44 | 2.80 |
| PAH | Acenaphthene | 83-32-9 | 19.39 | 2.60 |
| PAH | Dibenzofuran | 132-61-91 | 20.16 | 2.55 |
| PAH | 2,3,5-Trimethylnaphthalene | 2245-38-7 | 21.33 | 2.04 |
| PAH | Fluorene | 86-73-7 | 21.71 | 2.67 |
| PAH | 1-Methylfluorene | 1730-37-6 | 24.53 | 2.59 |
| PAH | 1,2,5,6-Tetramethylnaphthalene | 2131-43-3 | 25.21 | 2.27 |
| PAH | Dibenzothiophene | 132-65-0 | 25.31 | 3.29 |
| PAH | Phenanthrene | 85-01-8 | 25.97 | 3.26 |
| PAH | 4-Methyldibenzothiophene | 7372-88-5 | 27.48 | 3.04 |
| PAH | 1-Methylphenanthrene | 832-69-9 | 28.86 | 3.23 |
| PAH | 3,6-dimethylphenanthrene | 1576-67-6 | 30.69 | 2.87 |
| PAH | Fluoranthene | 206-44-0 | 31.34 | 3.66 |
| PAH | 1,2-Dimethylphenanthrene | 20291-79-2 | 31.91 | 3.24 |
| PAH | Pyrene | 129-00-0 | 32.24 | 3.97 |
| PAH | 2,6,9-Trimethylphenanthrene | 66271-32-7 | 33.47 | 2.79 |
| PAH | 1-Methylpyrene | 2381-21-7 | 34.87 | 3.91 |
| PAH | 1,2,6,9-Tetramethylphenanthrene | 204256-39-3 | 36.31 | 2.90 |
| PAH | Chrysene | 218-01-9 | 37.90 | 4.15 |
| PAH | Benz[a]anthracene | 56-55-3 | 37.98 | 4.25 |

| | | | | |
|---|---|---|---|---|
| PAH | 1-Methylchrysene | 3351-28-8 | 40.41 | 4.31 |
| PAH | Benzo[b]fluoranthene | 205-99-2 | 42.55 | 4.77 |
| PAH | Benzo[e]pyrene | 192-97-2 | 43.53 | 5.27 |
| PAH | Benzo[a]pyrene | 50-32-8 | 43.64 | 5.27 |
| PAH | Perylene | 198-55-0 | 43.99 | 5.45 |
| PAH | Indeno[1,2,3-cd]pyrene | 193-39-5 | 47.78 | 5.75 |
| PAH | Dibenz[ah]anthracene | 224-41-9 | 47.91 | 5.72 |
| PAH | Benzo[ghi]perylene | 191-24-2 | 48.61 | 0.31 |
| PBDEs | 2,4,4'-Tribromodiphenyl ether | 41318-75-6 | 34.76 | 3.41 |
| PBDEs | 2,2',4,4'-Tetrabromodiphenyl ether | 5436-43-1 | 38.91 | 3.83 |
| PBDEs | 2,3',4',6-Tetrabromodiphenyl ether | 189084-62-6 | 39.44 | 3.98 |
| PBDEs | 2,2',4,4',6-Pentabromodiphenyl ether | 189084-64-8 | 41.94 | 4.09 |
| PBDEs | 2,2',3,4,4'-Pentabromodiphenyl ether | 182346-21-0 | 42.81 | 4.19 |
| PBDEs | 2,2',4,4',5-Pentabromodiphenyl ether | 60348-60-9 | 44.21 | 4.87 |
| PBDEs | 2,2',4,4',5,5'-Hexabromodiphenyl ether | 68631-49-2 | 45.16 | 4.64 |
| PBDEs | 2,2',4,4',5,6'-Hexabromodiphenyl ether | 207122-15-4 | 46.31 | 4.67 |
| PCBs | 2,4,4’-Trichlorobiphenyl | 7012-37-5 | 27.97 | 2.48 |
| PCBs | 2,2’,5,5’-Tetrachlorobiphenyl | 35693-99-3 | 29.37 | 2.50 |
| PCBs | 2,2’,4,5,5’-Pentachlorobiphenyl | 37680-73-2 | 32.65 | 2.45 |
| PCBs | 2,3’,4,4’,5-Pentachlorobiphenyl | 31508-00-6 | 34.84 | 2.62 |
| PCBs | 2,2’,3,4,4’,5-Hexachlorobiphenyl | 35065-28-2 | 35.80 | 2.47 |
| PCBs | 2,2’,4,4’,5,5’-Hexachlorobiphenyl | 35065-27-1 | 36.66 | 2.79 |
| PCBs | 2,2’,3,4,4’,5,5’-Heptachlorobiphenyl | 35065-29-3 | 39.11 | 2.71 |
| Pesticide | Hexachlorobenzene | 118-74-1 | 24.68 | 2.03 |
| Phenol | 2,4-Dimethylphenol | 105-67-9 | 10.78 | 1.74 |
| Phenol | 4-Ethylphenol | 123-07-9 | 11.24 | 1.78 |
| Phenol | 3,5-Dimethylphenol | 108-68-9 | 11.38 | 1.73 |
| Phenol | 2,4,6-Trimethylphenol | 527-60-6 | 12.28 | 1.78 |
| Phenol | 2,3,5-Trimethylphenol | 697-82-5 | 14.11 | 1.85 |
| Phenol | 4-tert-Butylphenol | 98-54-4 | 14.69 | 1.74 |
| Phenol | 4-Isopropyl-3-methylphenol | 3228-02-2 | 15.71 | 1.85 |
| Phenol | 4-tert-Butyl-2-methylphenol | 98-27-1 | 16.48 | 1.67 |
| Phenol | 4-n-Pentylphenol | 14938-35-3 | 18.91 | 1.79 |
| Phenol | 2,6-Di-tert-butyl-4-methylphenol | 128-37-0 | 20.41 | 1.33 |
| Phenol | 2,6-Di-tert-butylphenol | 128-39-2 | 21.31 | 1.43 |
| Phenol | 4-Hexylphenol | 2446-69-7 | 21.32 | 1.79 |
| Phenol | 4-tert-Octylphenol | 140-66-9 | 22.46 | 1.67 |
| Phenol | 4-Heptylphenol | 1987-50-4 | 23.71 | 1.75 |
| Phenol | 4-n-Octylphenol | 1806-26-4 | 25.91 | 1.75 |
| Phenol | 4-n-Nonylphenol | 104-40-5 | 28.07 | 1.73 |
| Phthalates | Diethyl phthalate | 84-66-2 | 21.80 | 2.66 |
| Phthalates | Di-n-butylphthalate | 84-74-2 | 29.61 | 2.26 |
| Phthalates | Benzyl butyl phthalate | 85-68-7 | 36.24 | 3.28 |
| Phthalates | Bis(2-ethylhexyl)adipate | 103-23-1 | 37.71 | 1.25 |
| Phthalates | Di-n-octyl phthalate | 117-84-0 | 42.51 | 2.00 |

| | | | | |
|---|---|---|---|---|
| Plastic/rubber chemical | Benzothiazole | 95-16-9 | 12.52 | 3.03 |
| Plastic/rubber chemical | n-Cyclohexylformamide | 766-93-8 | 13.06 | 3.17 |
| Plastic/rubber chemical | Phthalide | 87-41-2 | 15.31 | 4.21 |
| Plastic/rubber chemical | 2,4,7,9-Tetramethyl-5-decyne-4,7-diol | 126-86-3 | 18.10 | 1.07 |
| Plastic/rubber chemical | Phthalimide | 85-41-6 | 18.18 | 4.25 |
| Plastic/rubber chemical | Methylparaben | 99-76-3 | 18.51 | 2.83 |
| Plastic/rubber chemical | 3-Methyl-2(3H)-Benzothiazolone | 2786-62-1 | 21.11 | 4.00 |
| Plastic/rubber chemical | 2-(Methylthio)benzothiazole | 615-22-5 | 21.92 | 3.32 |
| Plastic/rubber chemical | n-Butylbenzenesulfonamide | 3622-84-2 | 25.51 | 3.67 |
| Plastic/rubber chemical | Bisphenol A | 80-05-7 | 33.11 | 4.30 |
| Plastic/rubber chemical | Bumetrizole | 3896-11-5 | 39.77 | 2.46 |
| PPCP | Isoeugenol | 97-53-0 | 18.56 | 2.21 |
| PPCP | Benzophenone | 119-61-9 | 22.53 | 3.12 |
| PPCP | Atrazine | 1912-24-9 | 25.08 | 2.99 |
| PPCP | Caffeine | 58-08-2 | 26.62 | 4.84 |
| PPCP | Allethrin | 584-79-2 | 32.11 | 1.79 |
| PPCP | Triclosan | 3380-34-5 | 32.20 | 2.83 |
| PPCP | Fluconazole | 86386-73-4 | 32.41 | 3.83 |
| PPCP | 17β-Estradiol | 50-28-2 | 40.65 | 4.43 |
| PPCP | Praziquantel | 55268-74-1 | 44.96 | 5.27 |
| PPCP | Nicotine | 54-11-5 | 16.11 | 2.03 |
| Various | Decalin, cis | 91-17-8 | 9.96 | 0.91 |
| Various | Decalin, trans | 91-17-8 | 10.01 | 0.89 |
| Various | Methyldecalin | 2958-76-1 | 11.61 | 0.89 |
| Various | Pristane | 1921-70-6 | 25.48 | 0.53 |
| Various | Phytane | 638-36-8 | 27.62 | 0.59 |
| Various | 5@Androstane | 438-22-2 | 31.09 | 1.51 |
| Various | 17alpha (H) 21 beta (H)-Hopane | 13849-96-2 | 48.21 | 2.09 |
| Various | o-Terphenyl | 84-15-1 | 28.27 | 2.76 |
| Various | 1-Phenyldodecane | 123-01-3 | 28.68 | 1.14 |
| Various | 3,4-Fluorobenzamide | 85118-04-3 | 14.64 | 4.24 |
| Various | 1-Pyrenol | 5315-79-7 | 38.21 | 5.32 |

## Figure S1

(**a**) Motor activity profiles of zebrafish larvae exposed to WAF (9.8%-100%). The y-axis represents motor activity over time, while the x-axis covers 26 behavioral endpoints, including baseline activity, visual startle, and stimulus-induced responses. Gray traces represent control larvae Control larvae were incubated in Hanks' Balanced Salt Solution (HBSS, n = 70).

(**b**) Raincloud plots depicting motor activity differences at 26 behavioral endpoints for zebrafish larvae exposed to increasing WAF concentrations. The horizontal dotted line represents the median control habituation score, while adjusted p-values from a two-sample bootstrapping test are displayed above each plot.

## WAF (9.8% - 100%)

**a**

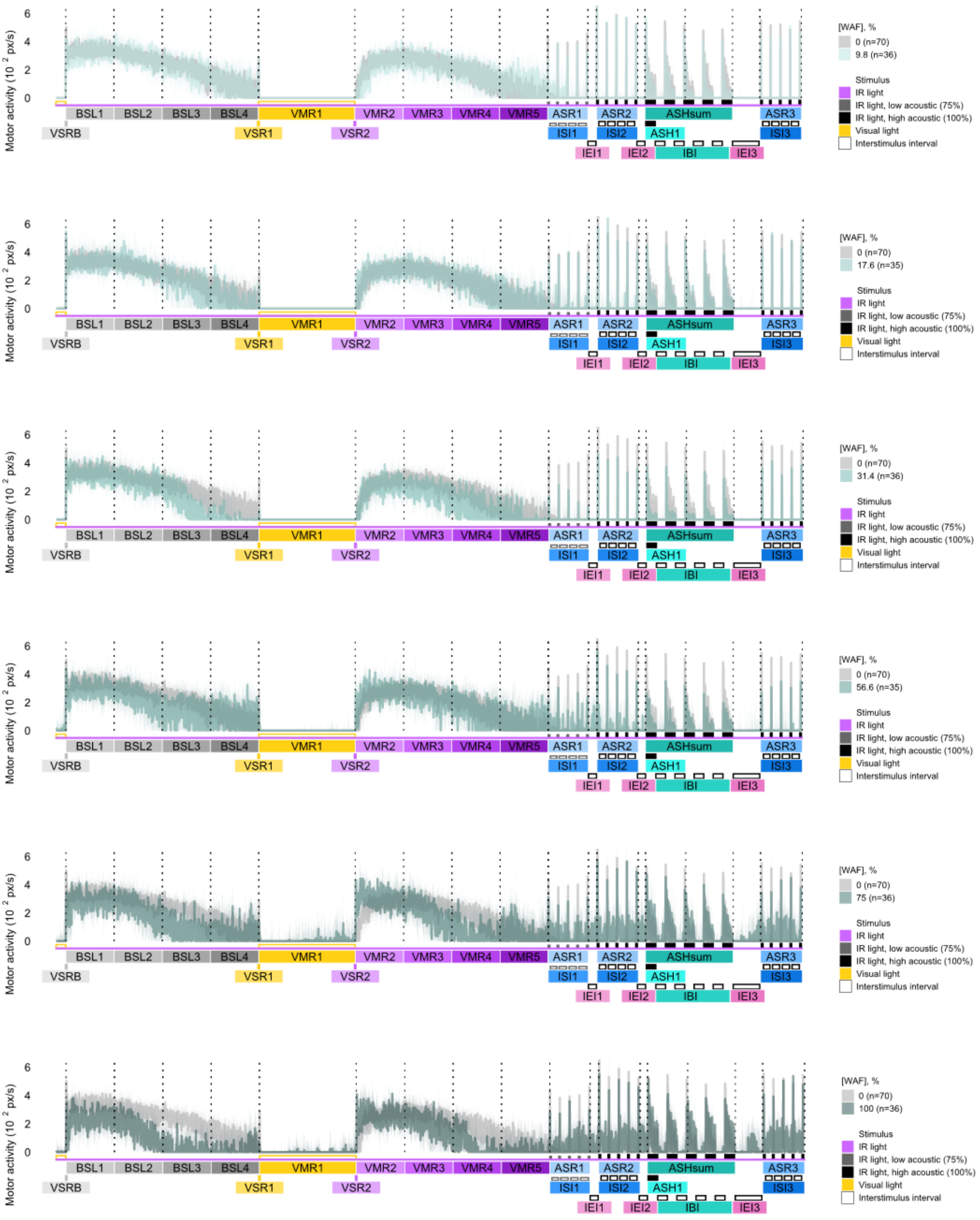

# WAF (9.8% - 100%)

**b**

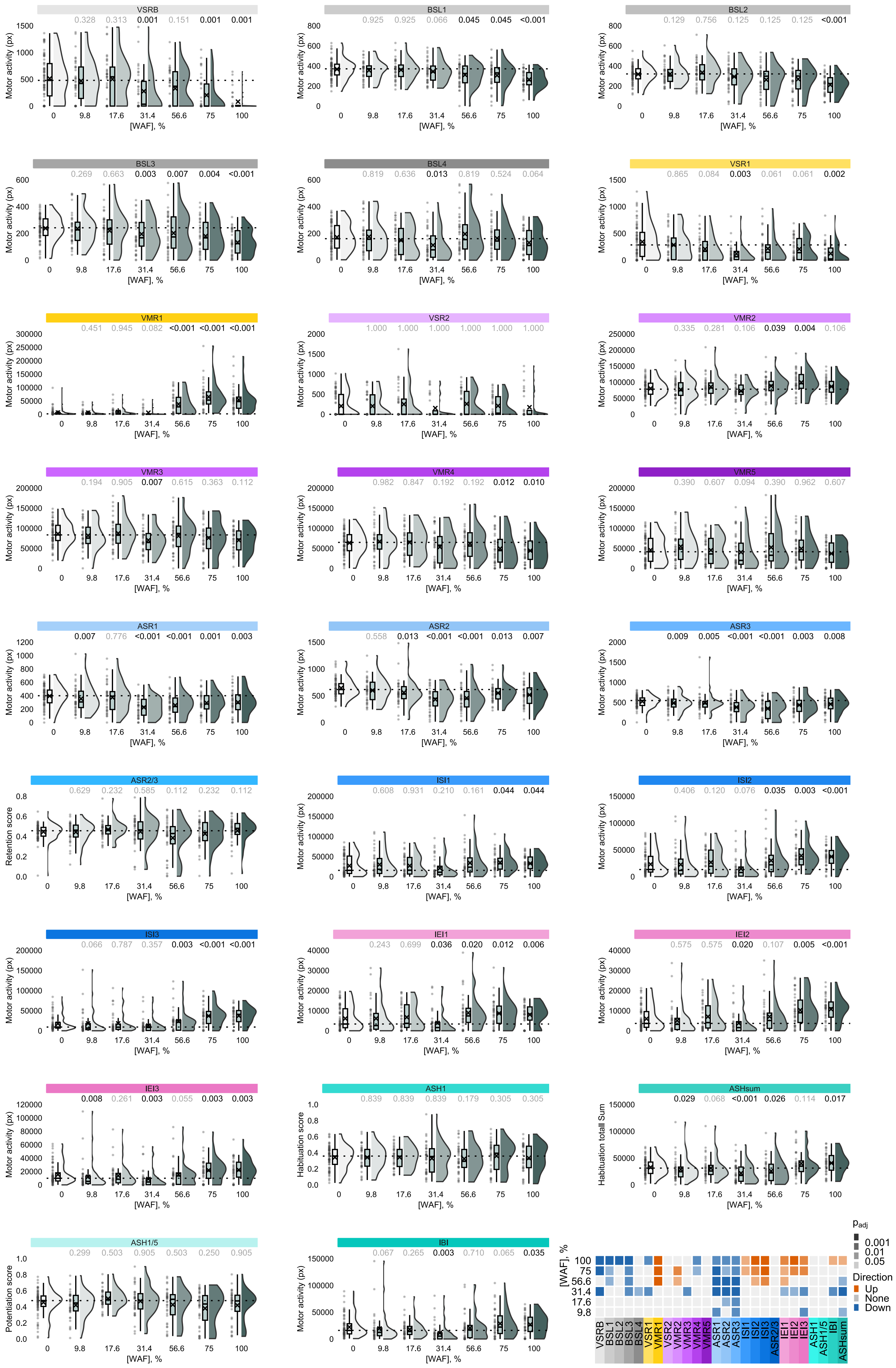

# Figure S2

Motor activity profiles and raincloud plots for zebrafish larvae exposed to the (**a**) WAF Fraction and its chemical components: (**b**) resins, (**c**) PAHs, (**d**) Sarutantes, (**e**) NAPs, and (**f**) MAH. The y-axis represents motor activity over time, while the x-axis spans 26 behavioral endpoints covering baseline, visual startle, vibrational motor responses, and stimulus-induced activity. Gray traces represent control larvae (n = 40-72), serving as blanks for each respective fraction.
Raincloud plots illustrate motor activity differences across the same 26 behavioral endpoints. The horizontal dotted line represents the median control habituation score, and adjusted p-values from a two-sample bootstrapping test are displayed above each plot.

**a** WAF Fraction

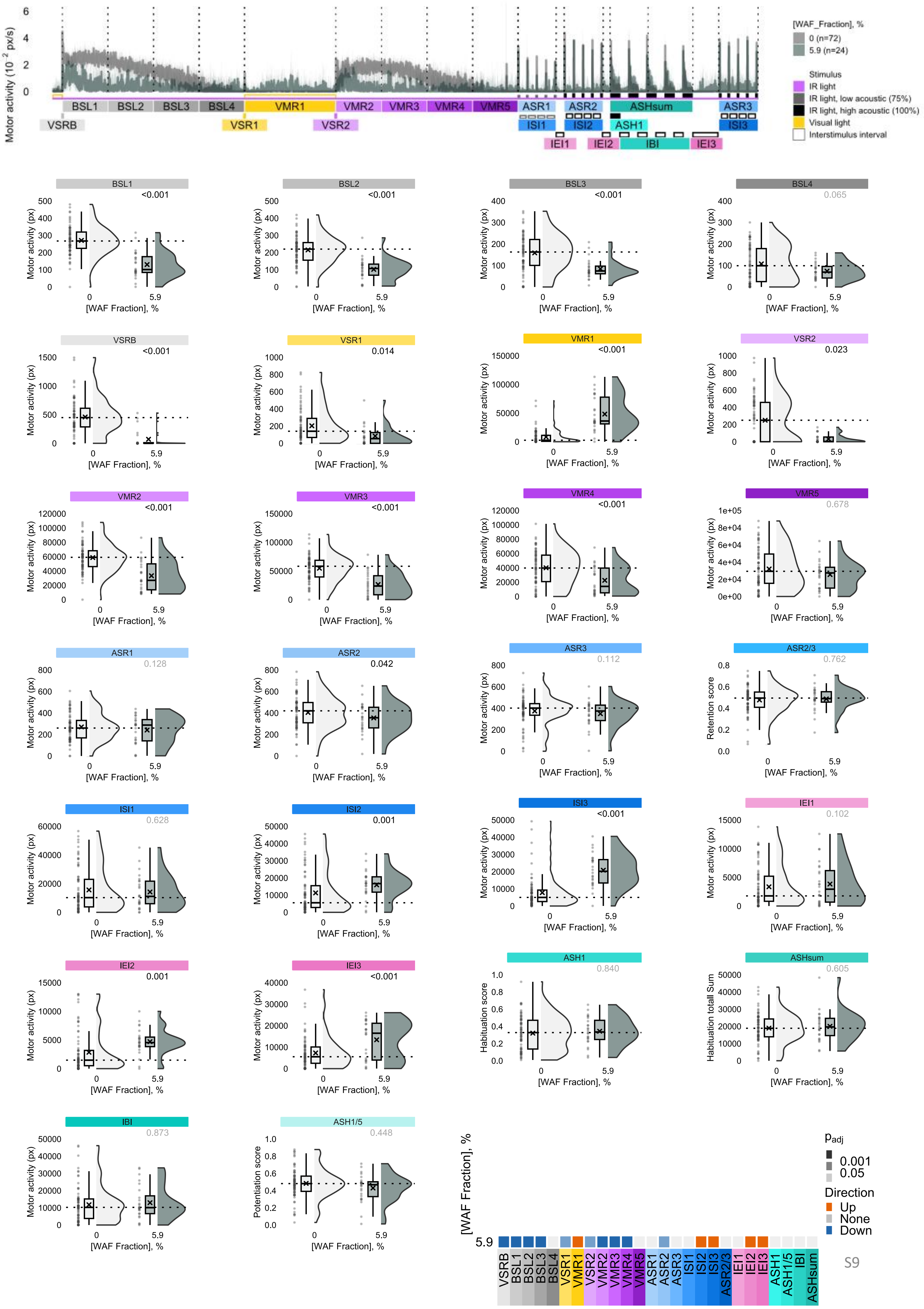

**b** **Resin Fraction**

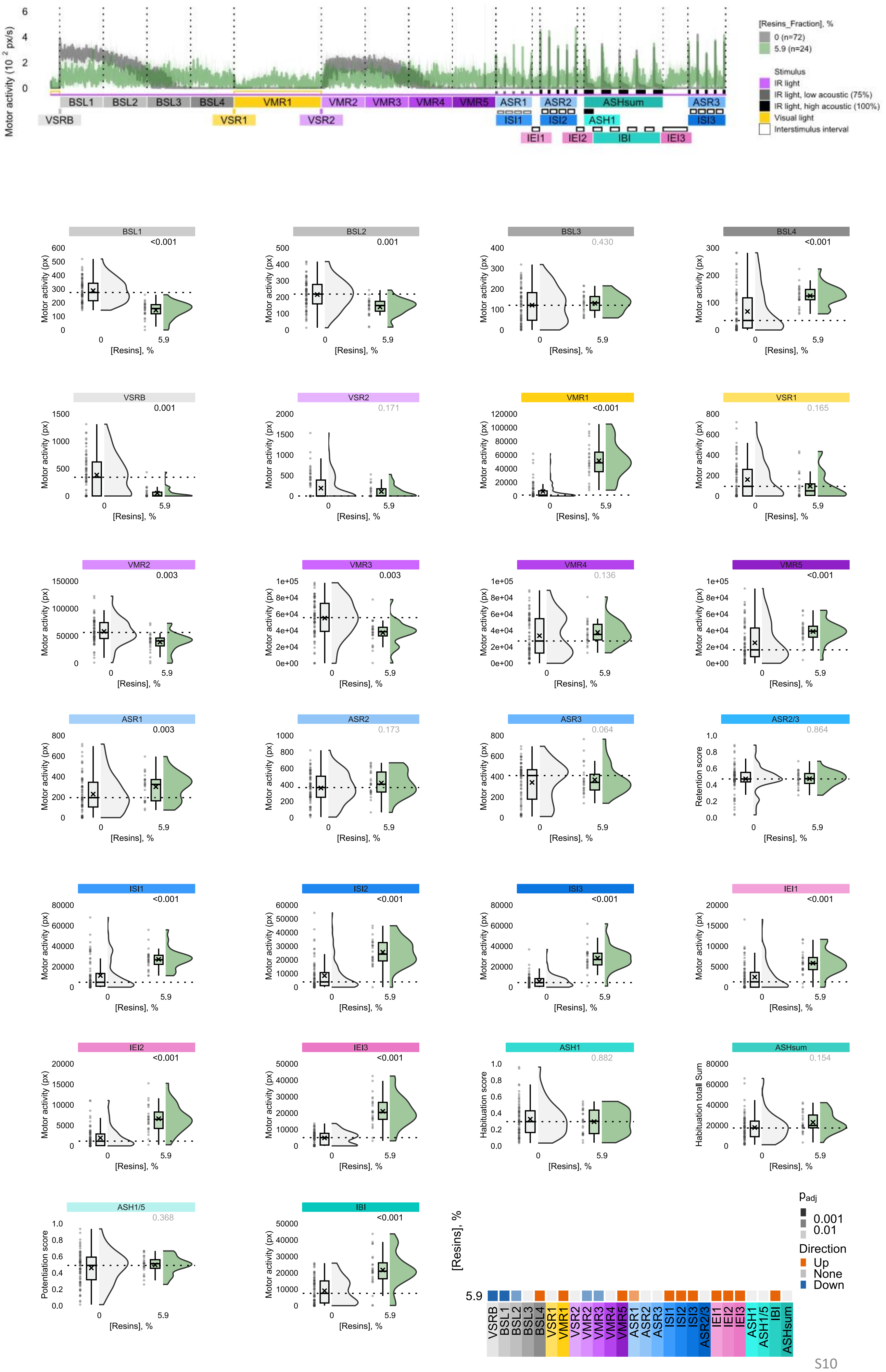

**c** **PAH Fraction**

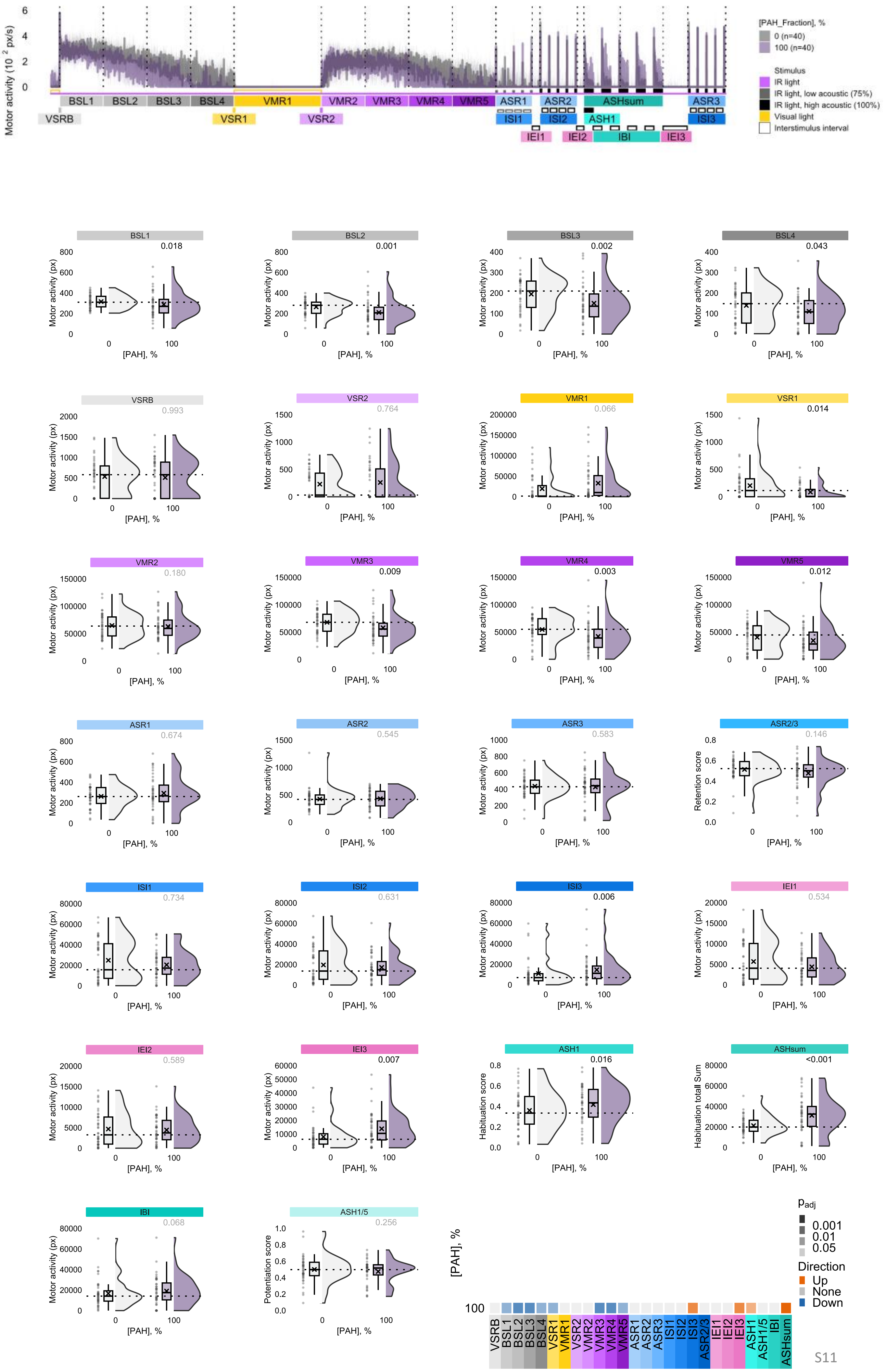

**d** **Saturates Fraction**

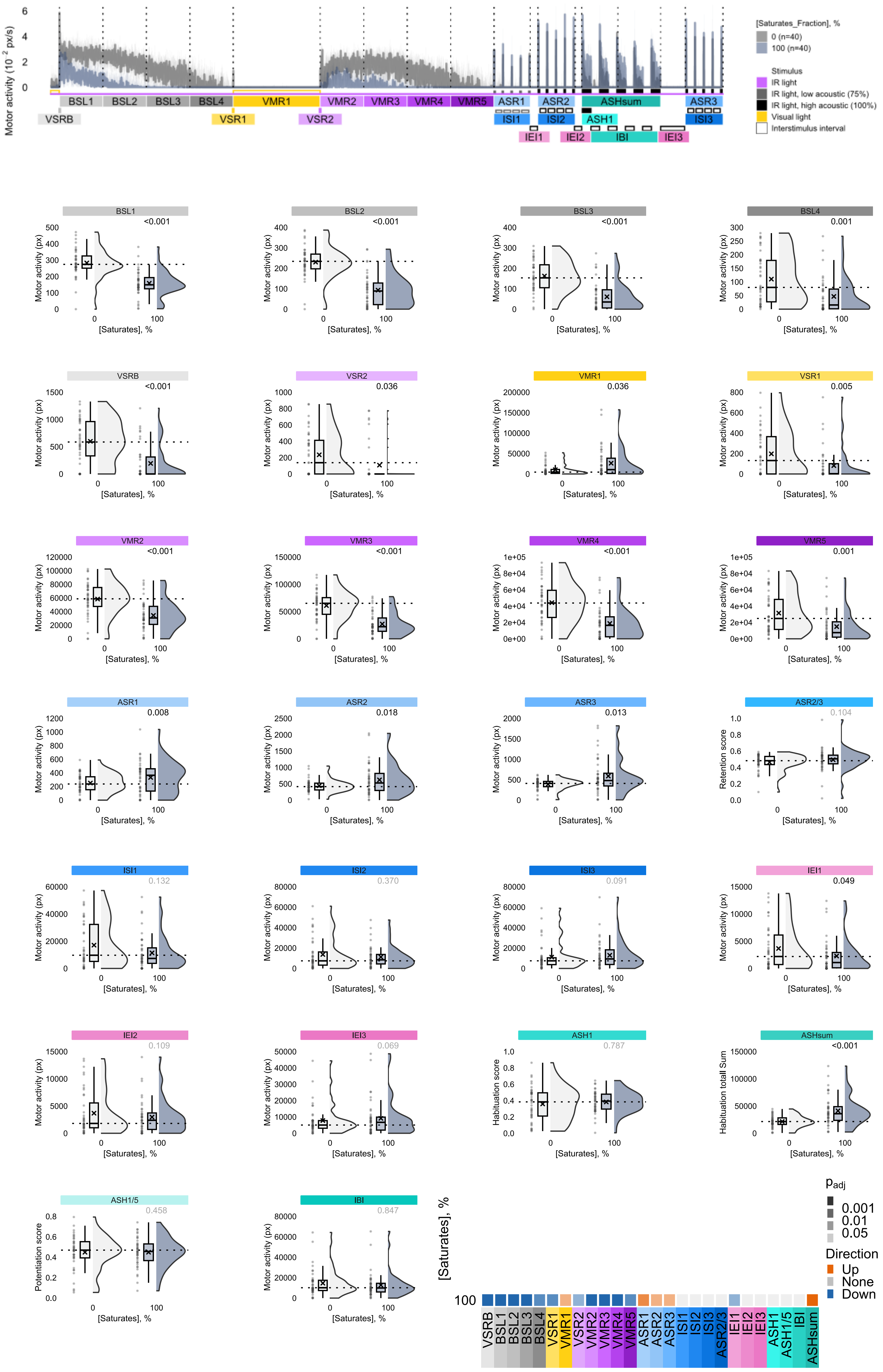

**e MAH Fraction**

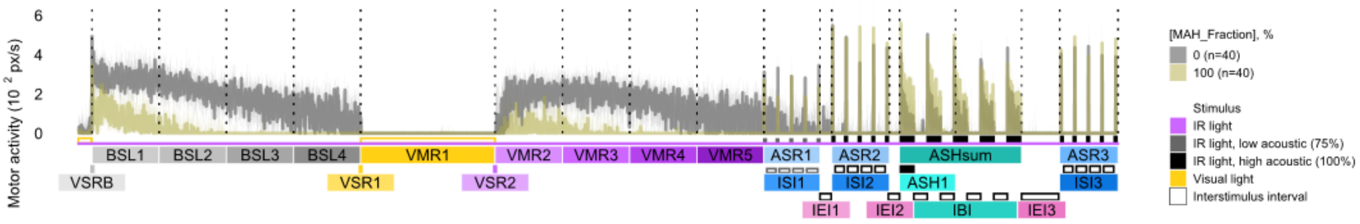


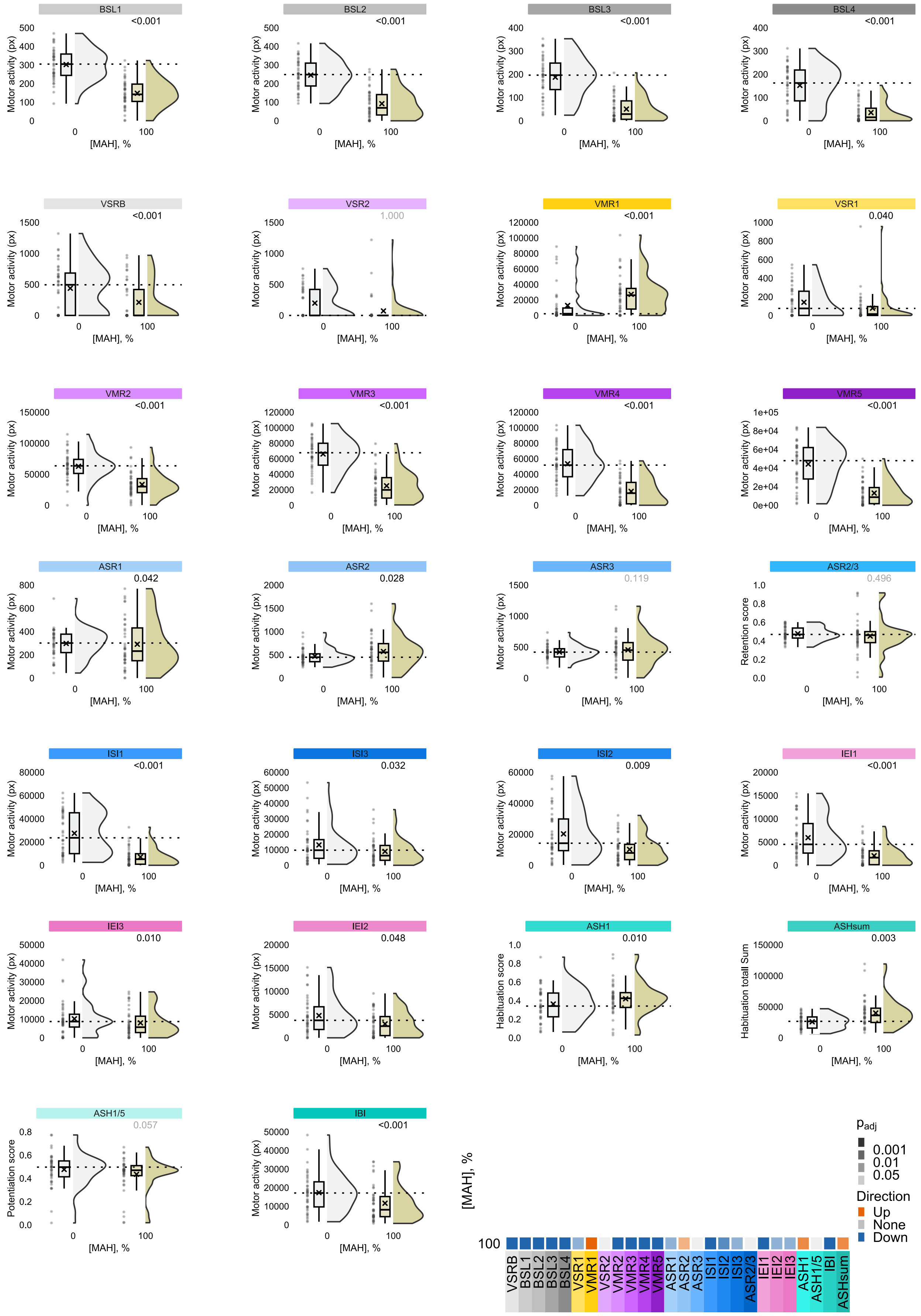

**f** NAP Fraction

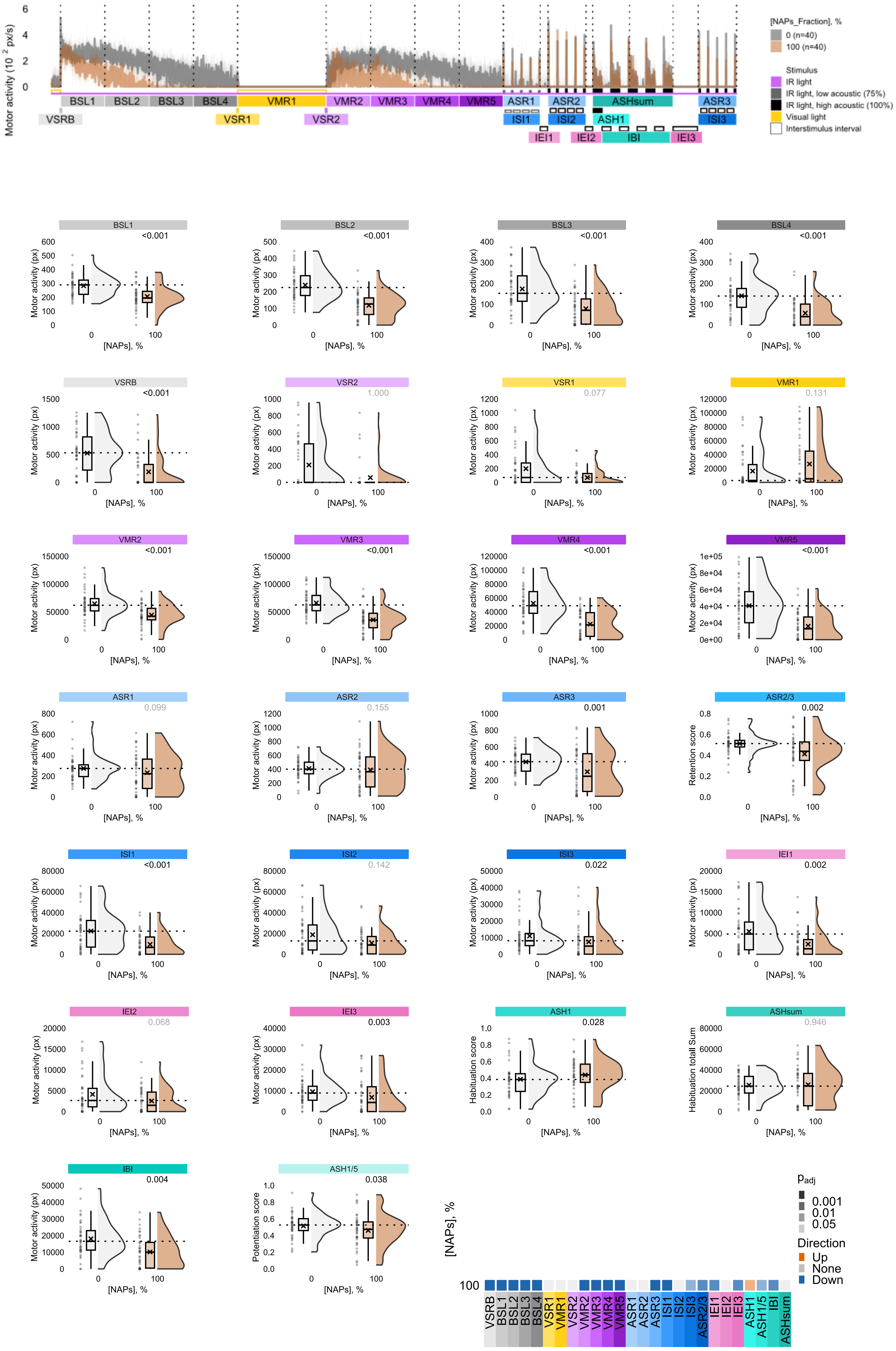

# Figure S3

(**a**) Hierarchical clustering of behavioral response profiles across a 26-endpoint assay battery (x-axis), comparing six concentrations of WAF and its chemical fractions—Saturates Fraction (blue), MAH Fraction, (yellow), NAPs Fraction (orange), PAHs Fraction (dark purple), and Resins Fraction (dark green). Controls varied by condition, with WAF exposures compared to HBSS-treated larvae and WAF fractions compared to their respective blanks (n=40-72). Heatmap showing SSMD effect sizes. Orange indicates increased activity, blue/purple indicates reduced activity (n = 33–44 larvae per group). (**b**) MDS representation of pairwise distances between behavioral profiles based on data shown in **a**. Fill color indicates deviation from the median control phenotype in the ASH1 endpoint where orange and blue reflect enhanced and reduce habituation, respectively. Median motor activity (y-axis) of vehicle control (0.4% DMSO, grey)

**a**

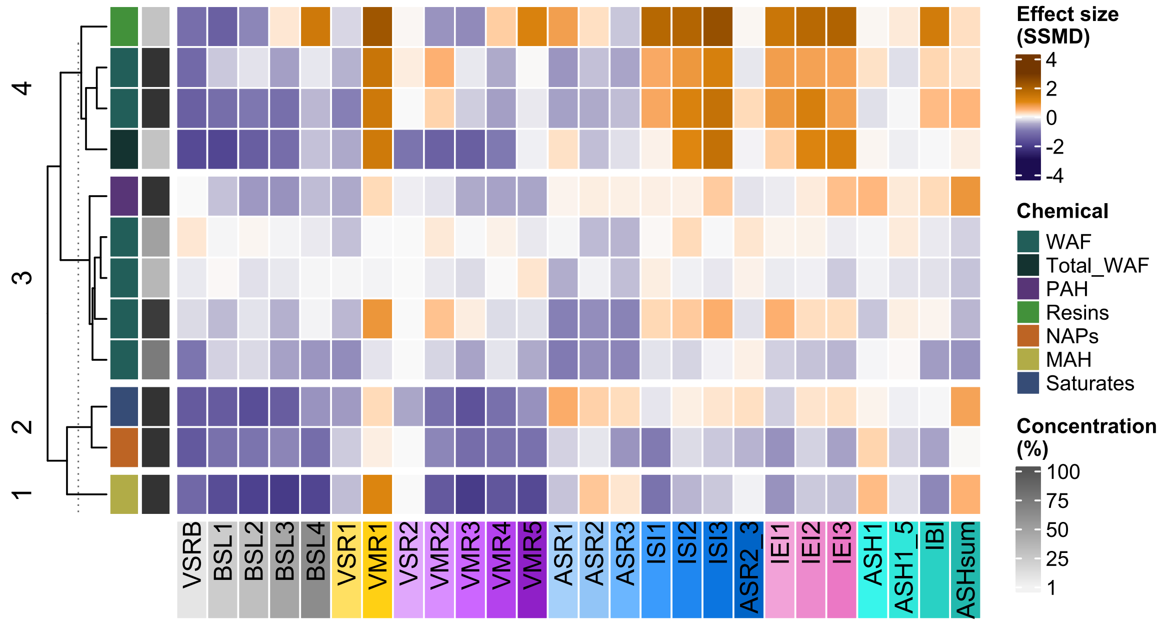


**b**

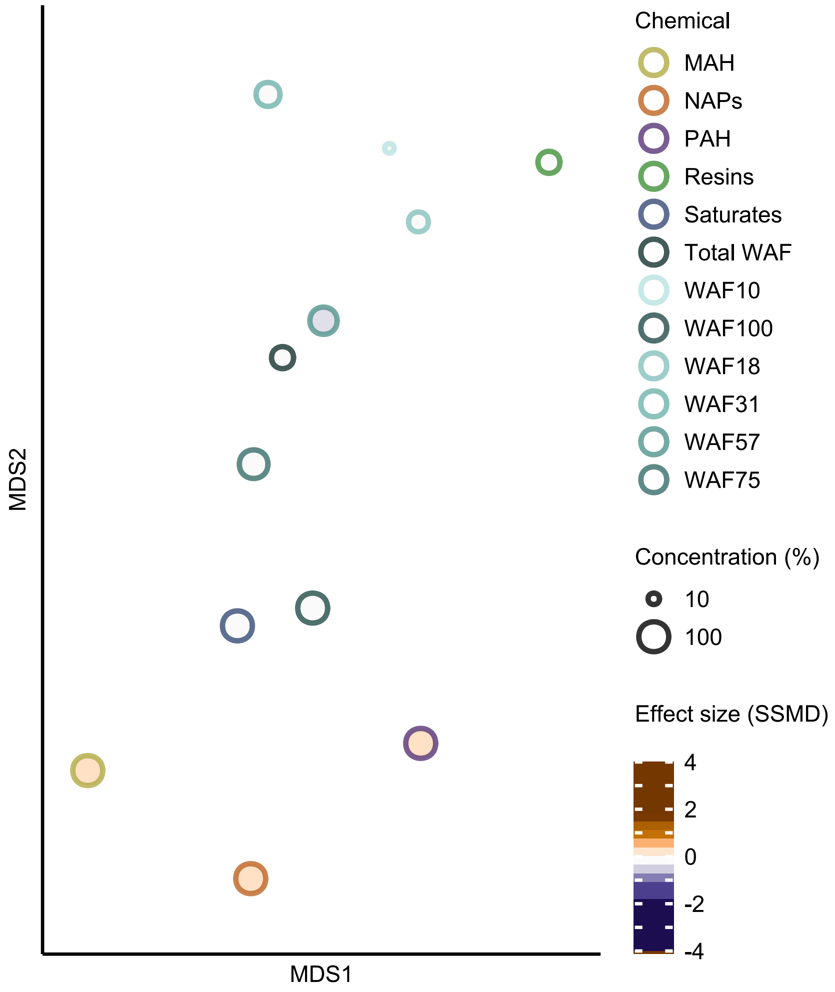

# Figure S4

(**a**) MAH fraction, (**b**) Saturate and (**c**) NAP fraction of WAF based on GC×GC-MS analysis. The x-axis indicates the percentage composition of every compound with respect to the whole PAH or Resin fraction (%), and the y-axis reports the chemical structures that were found.

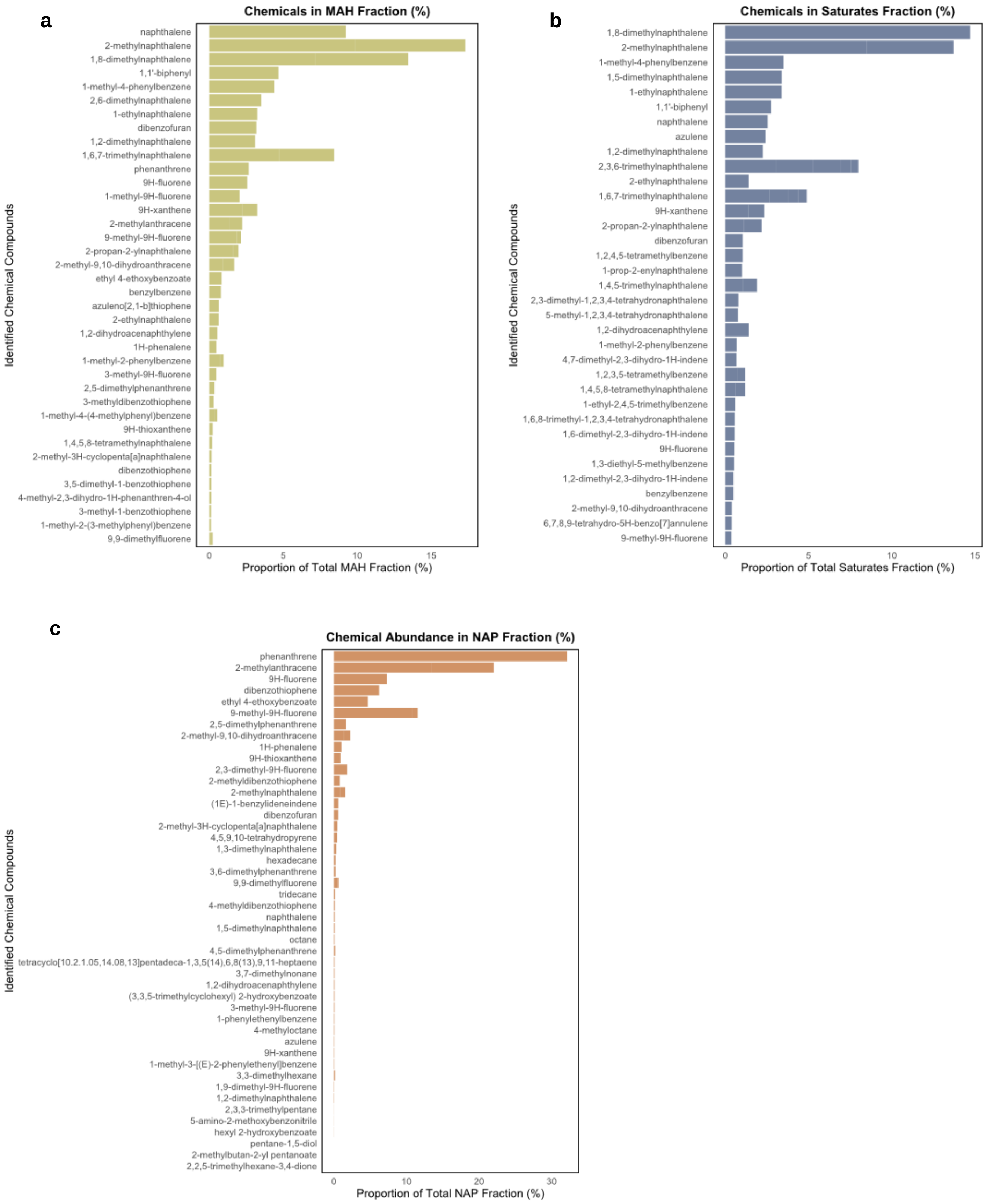

**Table S2|** Identified and tested individual chemicals for PAH and Resin fractions

| Fraction | Structure | Chemical | CAS number | DTXSID | SMILES | Supplier | Purity (%) | MW (g/mol) | Log P | Rt 1D | Rt 2D | RI | WAF Fraction | Relative Composition (%) |
|---|---|---|---|---|---|---|---|---|---|---|---|---|---|---|
| PAH | 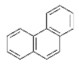 | Phenanthrene | 85-01-8 | DTXSID6024254 | C1=CC2=C(C=C1)C1=C(C=CC=C1)C=C2 | MCE | 99.6 | 178.234 | 4.5 | 25.96 | 3.81 | 1733 | 4175927 | 50 |
| | 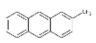 | 2-methylanthracene | 613-12-7 | DTXSID8060616 | CC1=CC2=CC3=CC=CC=C3C=C2C=C1 | TH Geyer Sigma-Aldrich | 98 | 192.261 | 5.1 | 28.9 | 3.78 | 1876 | 1218044 | 15 |
| | 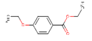 | 4-ethoxy-, ethyl ester | 23676-09-7 | DTXSID6073443 | CCOC(=O)C1=CC=C(OCC)C=C1 | TH Geyer Chem Pur | 97 | 194.23 | 3.2 | 20.44 | 2.4 | 1490 | 896681 | 11 |
| Resin | 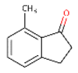 | 7-methyl-2,3-dihydroinden-1-one | 39627-61-7 | DTXSID60343420 | CC1=C2C(=O)CCC2=CC=C1 | Merck Sigma-Aldrich | 98 | 146.189 | 2 | 17.25 | 3.39 | 1364 | 18517107 | 4.36 |
| | 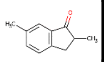 | 2,6-Dimethyl-2,3-dihydro-1H-inden-1-one | 66309-83-9 | DTXSID40469096 | C1CC2=C(C=C(C)C=C2)C1=O | TH Geyer BLDpharm | >98.0 | 160.216 | 2.6 | 17.98 | 3.00 | 1392 | 10277606 | 2.42 |
| | 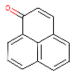 | 1H-phenalen-1-one | 548-39-0 | DTXSID20203278 | O=C1C=CC2=CC=CC3=C2C1=CC=C3 | Achemblock | 95 | 180.206 | 3.2 | 24.91 | 4.08 | 1684 | 11687732 | 2.75 |

**Table S3|** Toxicity Summary of the Top 10 Resin Fraction Compounds. Chemical structures, SMILES, and summary of known or potential (neuro)toxicity are provided based on publicly available data (PubChem, ECHA, EPA, CompTox, literature). “No data found” indicates absence of compound-specific information.

| Chemical | CAS | Structure | Smiles | Known (Neuro)Toxicity | Reference |
|---|---|---|---|---|---|
| 9,10-dimethyl-1,2,3,4-tetrahydroanthracene | 94573-50-9 | 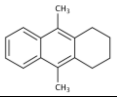 | CC1=C2CCCCC2=C(C)C2=CC=CC=C12 | No data found | |
| 4,7-dimethyl-2,3-dihydroinden-1-one | 5037-60-5 | 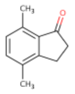 | CC1=C2CCC(=O)C2=C(C=C1)C | No data found | |
| 7-methyl-2,3-dihydsroinden-1-one | 39627-61-7 | 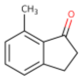 | CC1=C2C(=CC=C1)CCC2=O | No specific neurotoxicity or neuroactivity data. The substance is classified with general hazards (skin/eye irritation, respiratory effects) in safety data sheets, but no neural endpoints (behavior, neurotransmitters, developmental neurotoxicity, etc.) were identified. | National Center for Biotechnology Information (2025). PubChem Compound Summary for CID 589669, 7-methyl-2,3-dihydro-1H-inden-1-one. Retrieved October 14, 2025 from https://pubchem.ncbi.nlm.nih.gov/compound/7-methyl-2_3-dihydro-1H-inden-1-one. |
| 3,4,7-trimethyl-2,3-dihydroinden-1-one | 35322-84-0 | 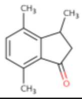 | CC1CC(=O)C2=C(C=CC(=C12)C)C | No data found | |
| 10H-anthracen-9-one | 90-44-8 | 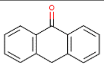 | C1C2=CC=CC=C2C(=O)C3=CC=CC=C31 | No specific neurotoxicity data found | |
| 1-(4-benzylphenyl)ethanone | 782-92-3 | 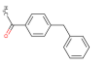 | CC(=O)C1=CC=C(C=C1)CC2=CC=CC=C2 | No data found | |
| 2,6-dimethyl-2,3-dihydroinden-1-one | 66309-83-9 | 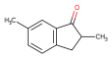 | CC1CC2=C(C1=O)C=C(C=C2)C | No direct neurotoxicity data found. Acute toxicity to Daphnia magna (48 h $EC_{50}$ = 10.5 mg $L^{-1}$) indicates potential neuromuscular interference in aquatic species (ECHA 2025). | ECHA registration dossier — *Short-term toxicity to aquatic invertebrates (Daphnia magna)*, OECD 202 guideline study, $EC_{50}$ = 10.5 mg/L. https://echa.europa.eu/en/registration-dossier/-/registered-dossier/34502/6/2/4 |
| dibenzofuran | 132-64-9 | 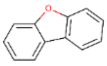 | O1C2=CC=CC=C2C2=C1C=CC=C2 | No specific neurotoxicity data available. According to EPA, no acute, chronic, reproductive, or developmental toxicity data exist for this compound, and no RfD or RfC values are established (IRIS). However, dibenzofuran is persistent, bioaccumulative, and toxic to aquatic organisms, suggesting potential for chronic environmental effects. | • U.S. EPA (1999). *Health Effects Assessment for Dibenzofuran*. EPA/600/8-88/026. Environmental Criteria and Assessment Office, Cincinnati, OH.<br>• CompTox Chemical Dashboard entry (DTXSID2021993): https://comptox.epa.gov/dashboard/chemical/executive-summary/DTXSID2021993 |
| 9,9-dimethylxanthene | 19814-75-6 | 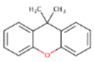 | CC1(C2=CC=CC=C2OC3=CC=CC=C31)C | No data found | |
| phenalen-1-one | 548-39-0 | 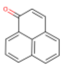 | O=C1C=CC2=CC=CC3=C2C1=CC=C3 | No data found | |

# Figure S5

Motor activity profiles of zebrafish larvae exposed to representative compounds from the PAH fraction including (**a**) phenanthrene, (**b**) 2-methylanthracene, and (**c**) ethyl 4-ethoxybenzoate. The y-axis represents motor activity over time, while the x-axis covers 26 behavioral endpoints, including baseline activity, visual startle, and stimulus-induced responses. Gray traces represent control larvae Control larvae were incubated in Hanks' Balanced Salt Solution (HBSS, n = 72). Raincloud of the same compounds plots depicting motor activity differences at 26 behavioral endpoints for zebrafish larvae exposed to increasing WAF concentrations. The horizontal dotted line represents the median control habituation score, while adjusted p-values from a two-sample bootstrapping test are displayed above each plot.

## a Phenanthrene

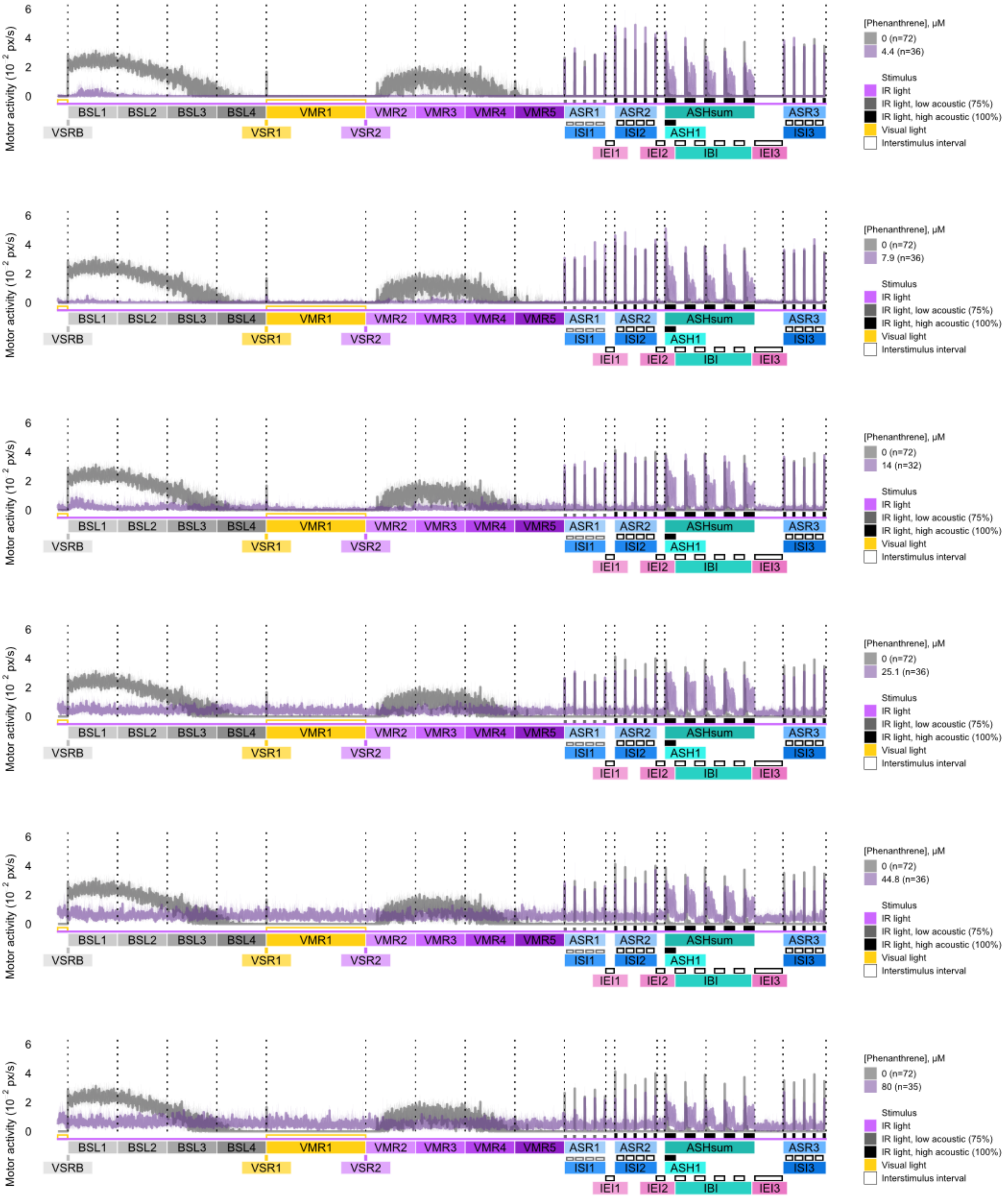

**a** **Phenanthrene**

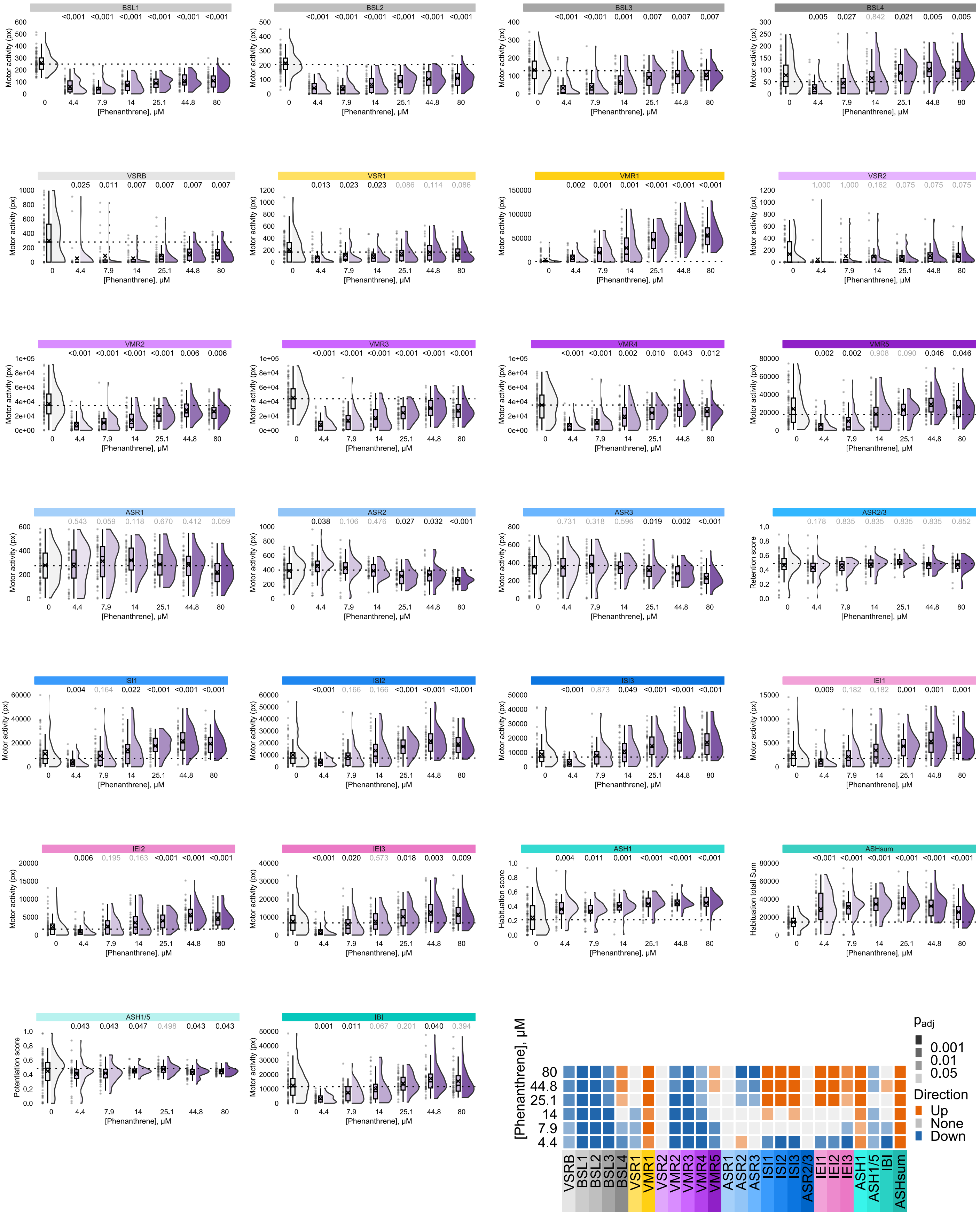

**b** **2-Methylanthracene**

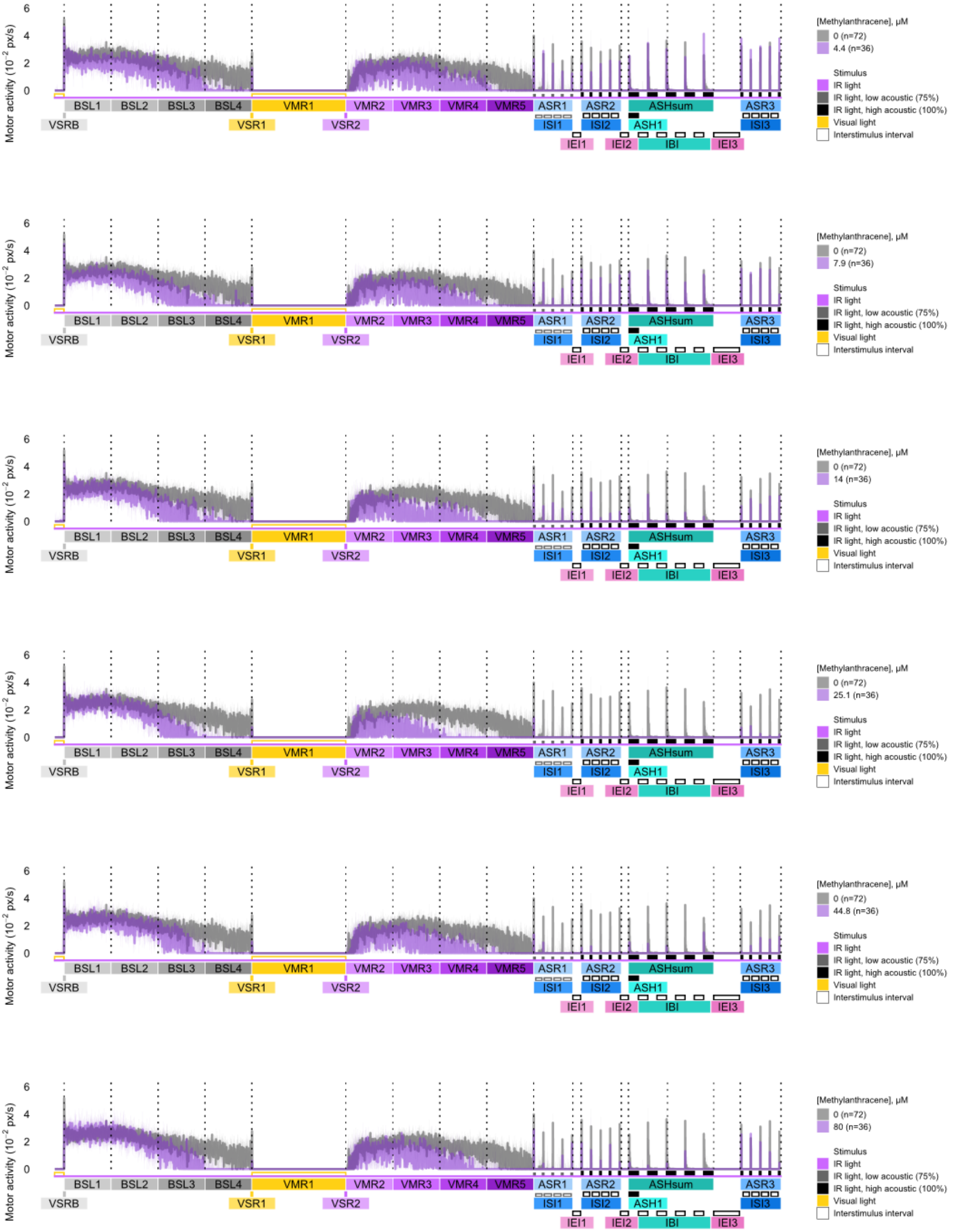

**c** **Ethyl 4-ethoxybenzoate**

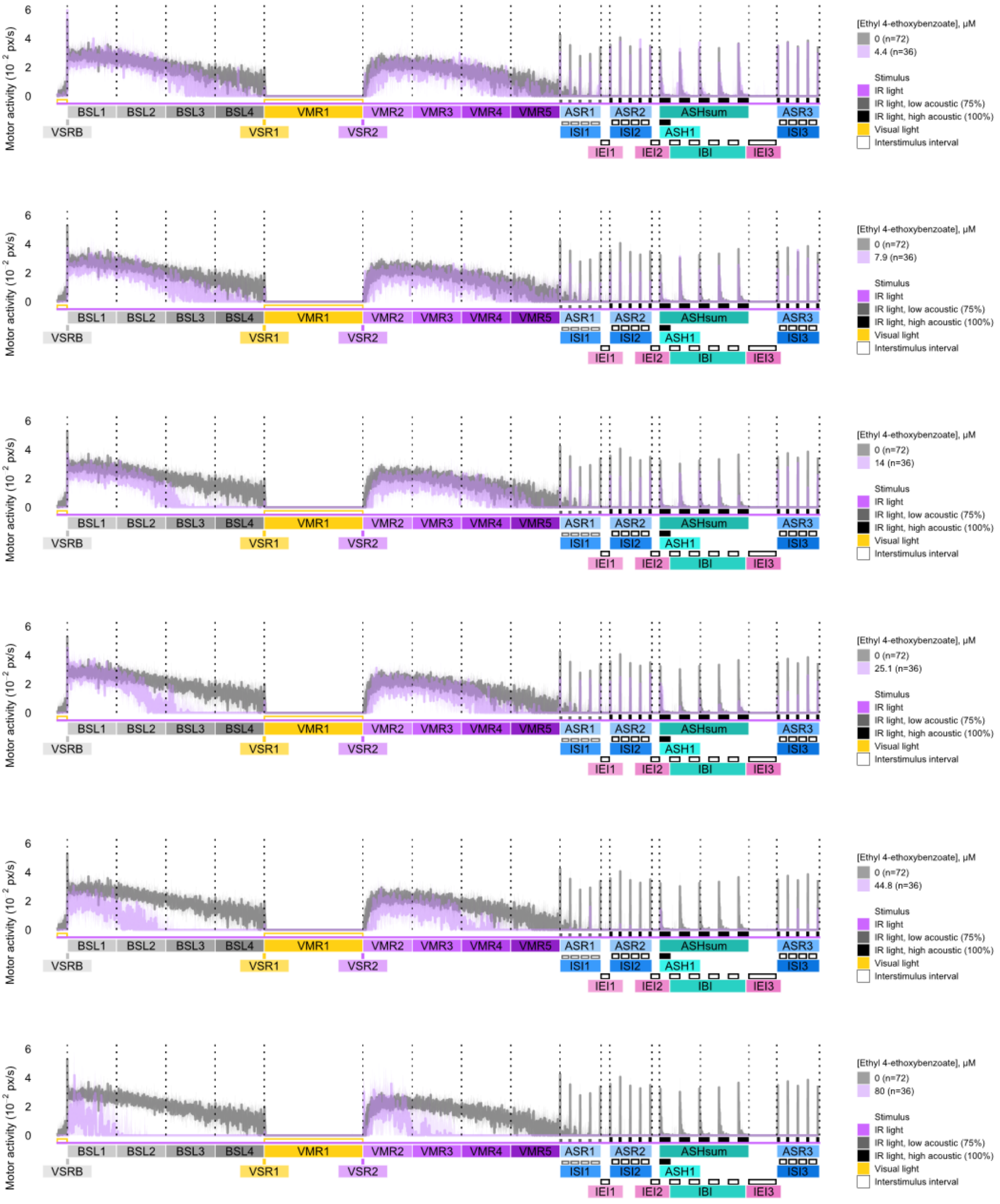

## c Ethyl 4-ethoxybenzoate

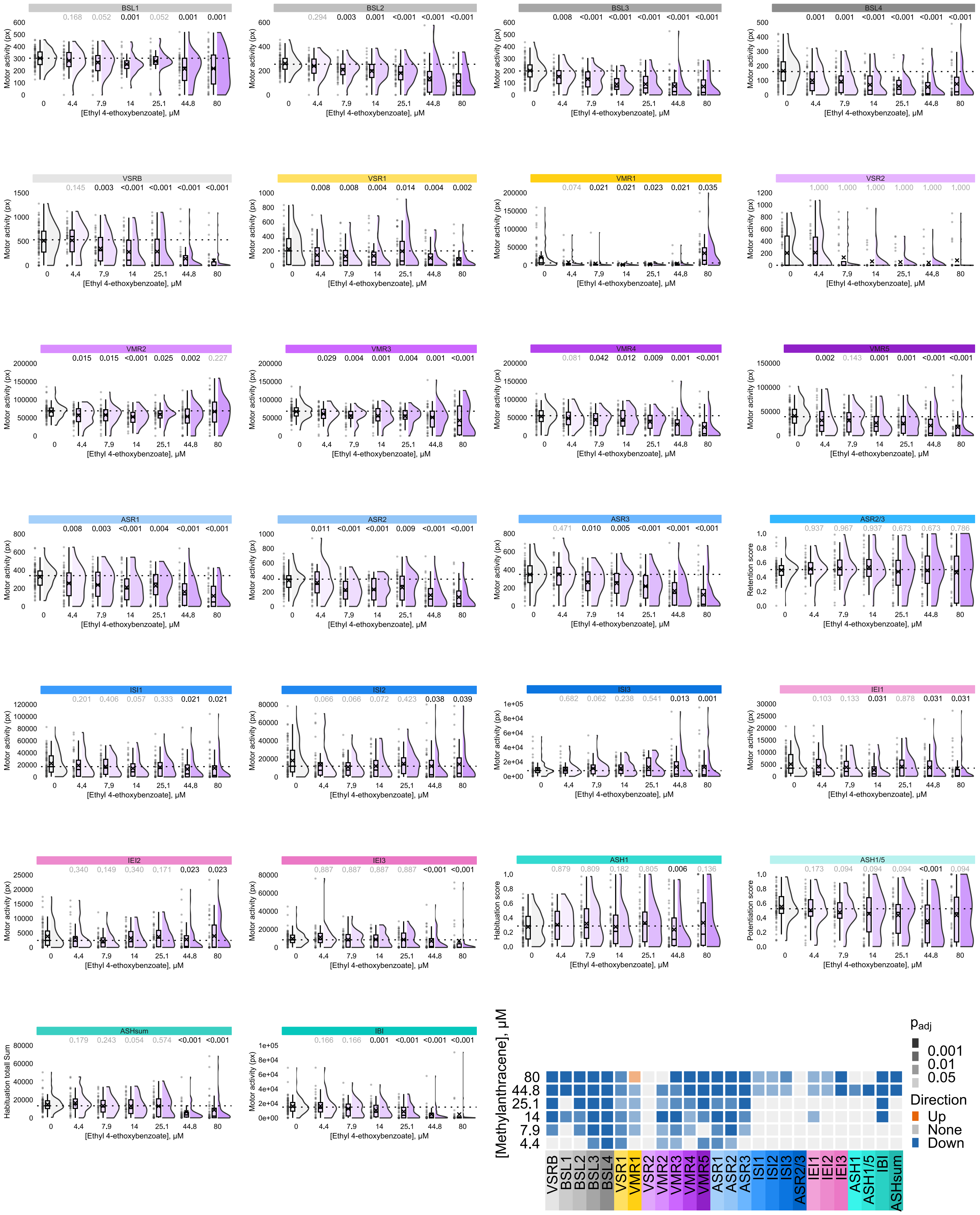

# Figure S6

Motor activity profiles of zebrafish larvae exposed to representative compounds from the resin fraction including (**a**) 2,6-Dimethyl-2,3-dihydro-1H-inden-1-one, (**b**) 7-methyl-2,3-dihydroinden-1-one, and (**c**) 1H-phenalen-1-one. The y-axis represents motor activity over time, while the x-axis covers 26 behavioral endpoints, including baseline activity, visual startle, and stimulus-induced responses. Gray traces represent control larvae Control larvae were incubated in Hanks' Balanced Salt Solution (HBSS, n = 72).

Raincloud of the same compounds plots depicting motor activity differences at 26 behavioral endpoints for zebrafish larvae exposed to increasing WAF concentrations. The horizontal dotted line represents the median control habituation score, while adjusted p-values from a two-sample bootstrapping test are displayed above each plot.

## a 2,6-Dimethyl-2,3-dihydro-1H-inden-1-one

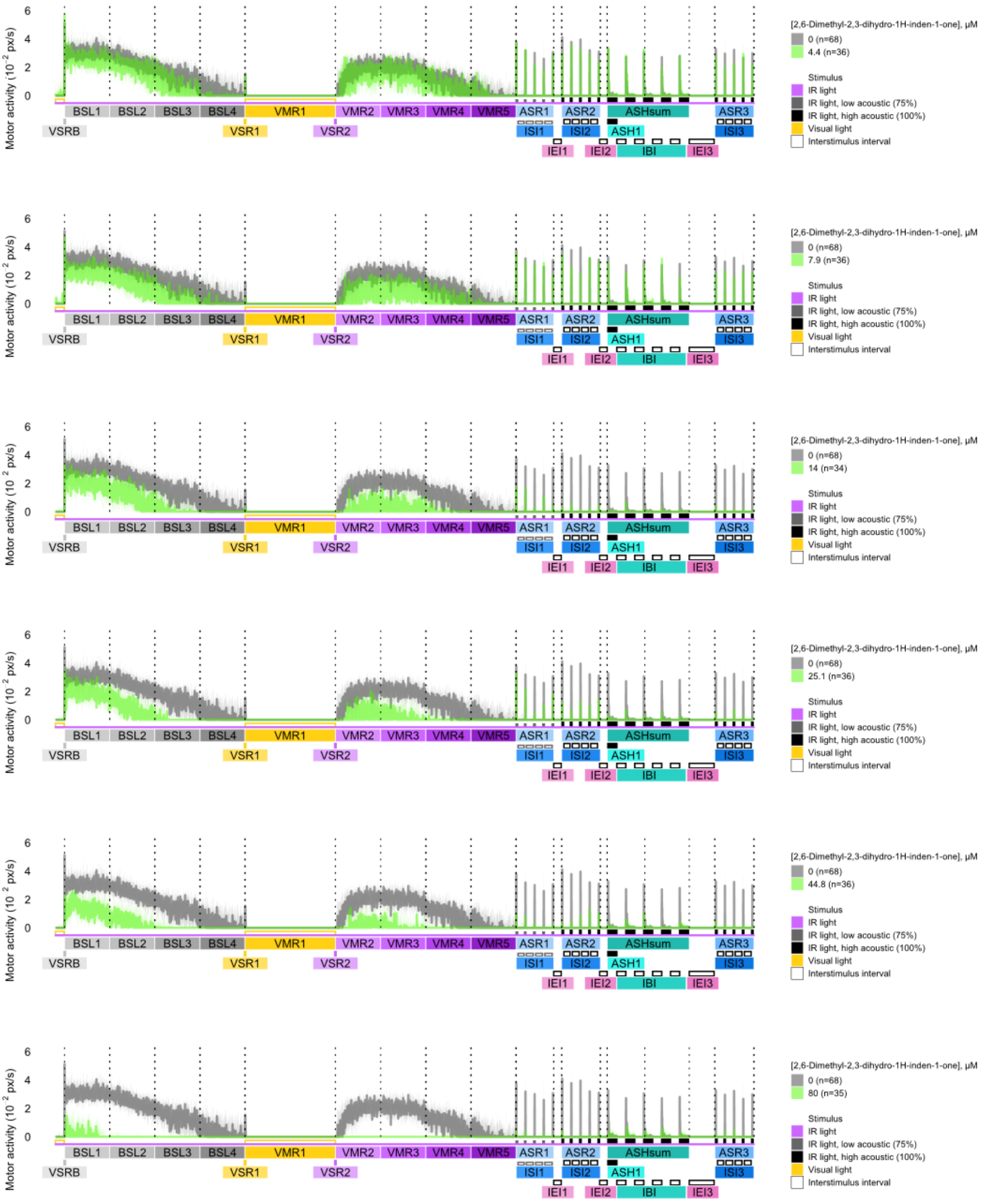

## a 2,6-Dimethyl-2,3-dihydro-1H-inden-1-one

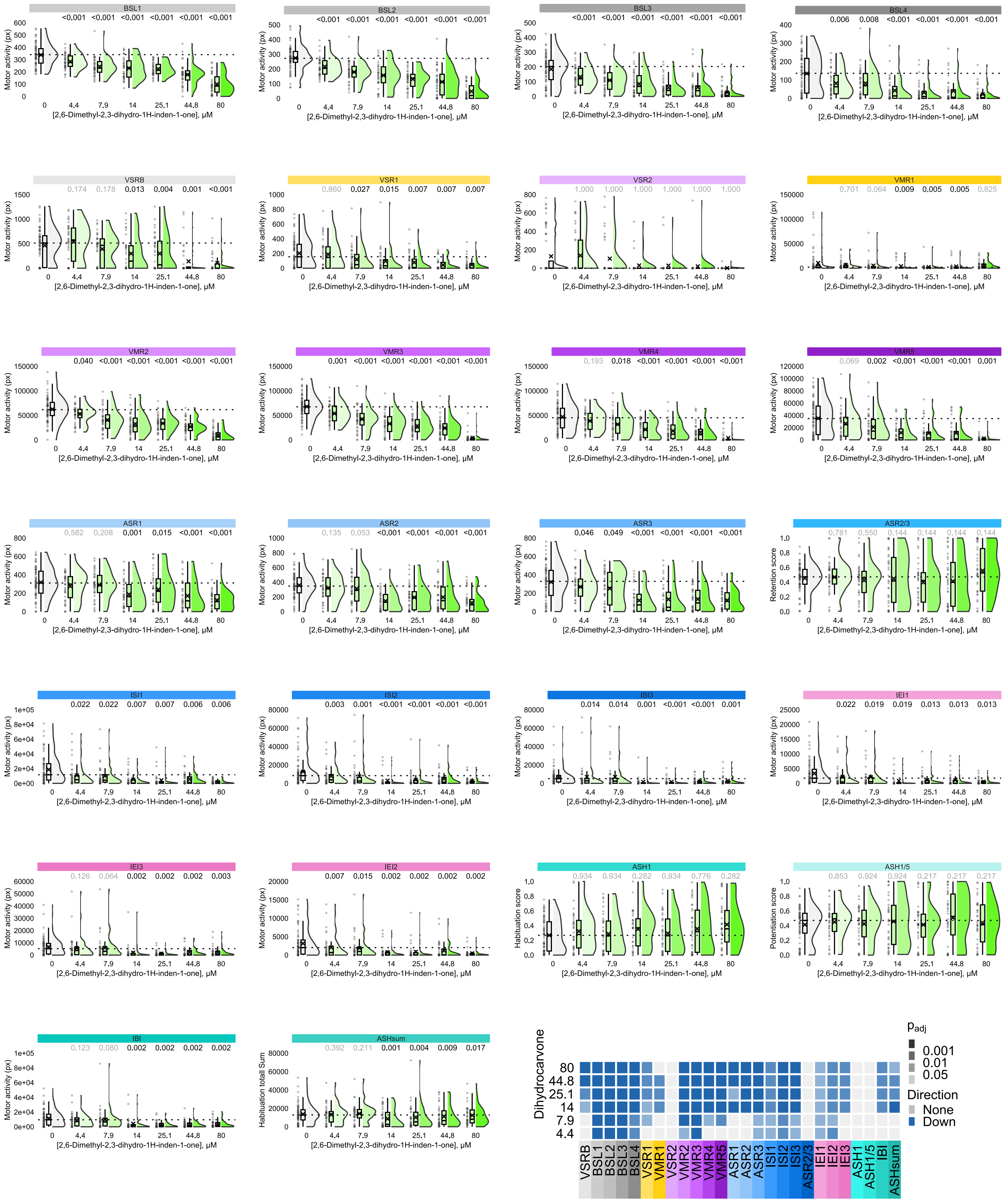

**b** **7-methyl-2,3-dihydroinden-1-one**

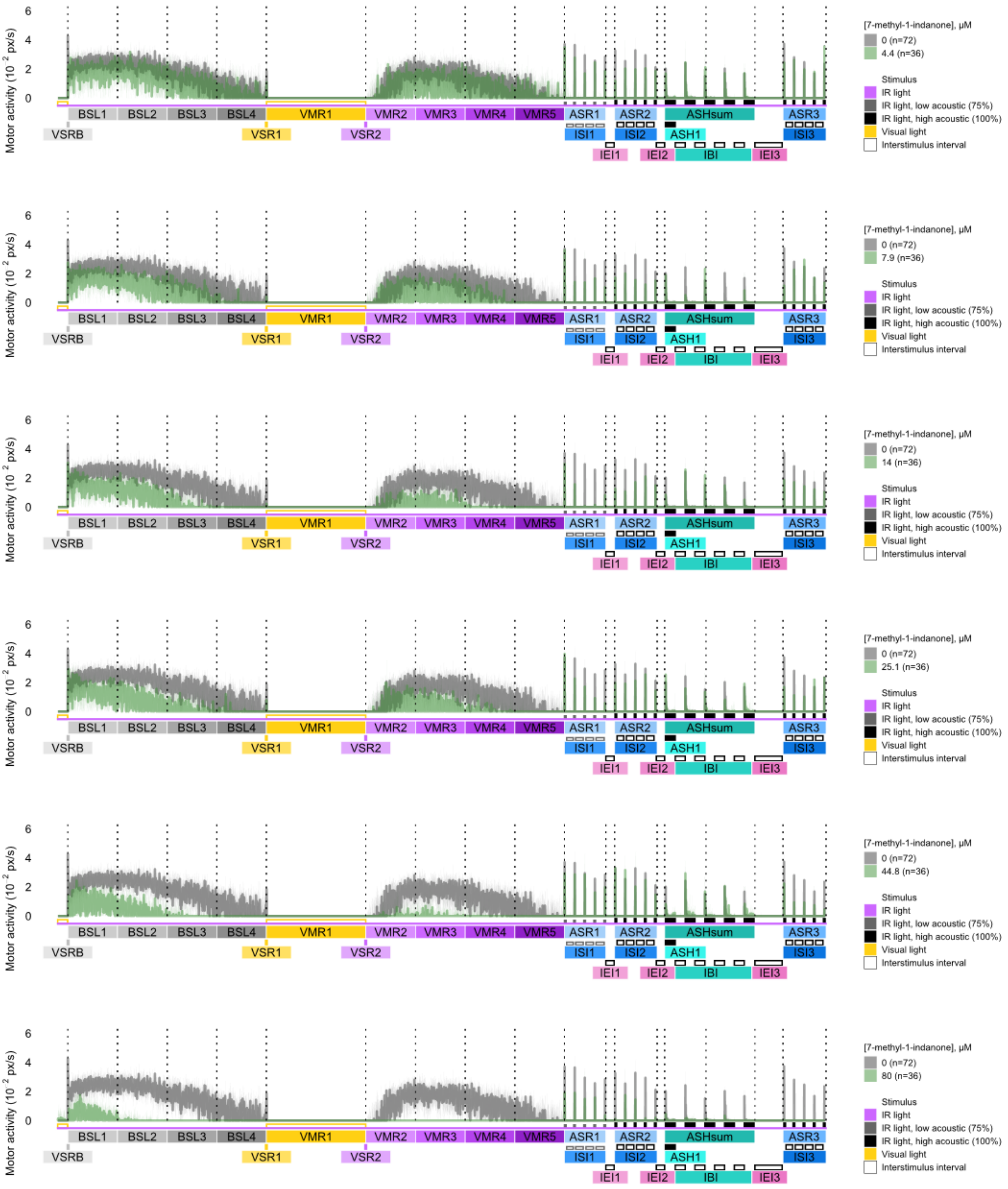

## b 7-methyl-2,3-dihydroinden-1-one

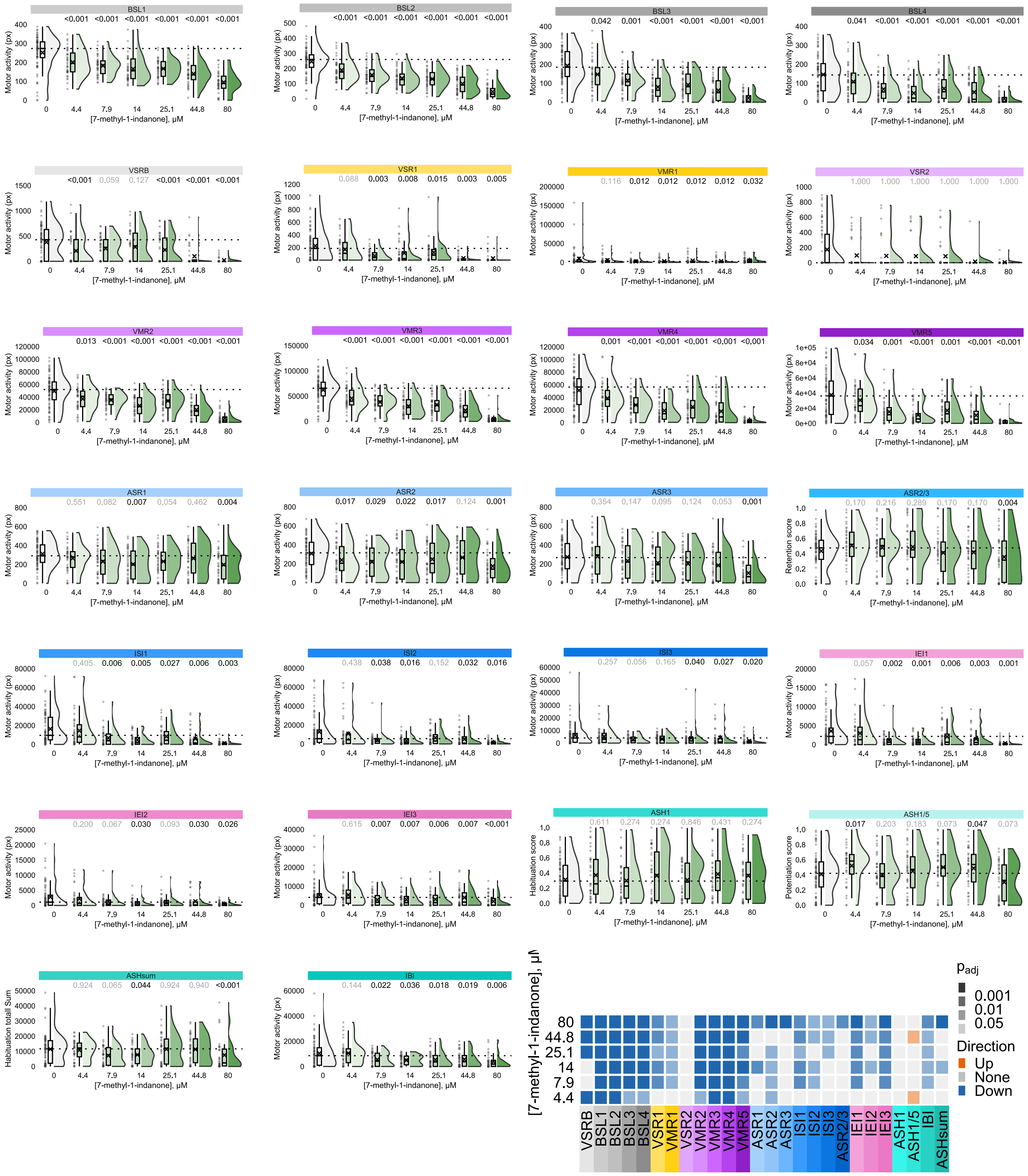

**c 1H-phenalen-1-one**

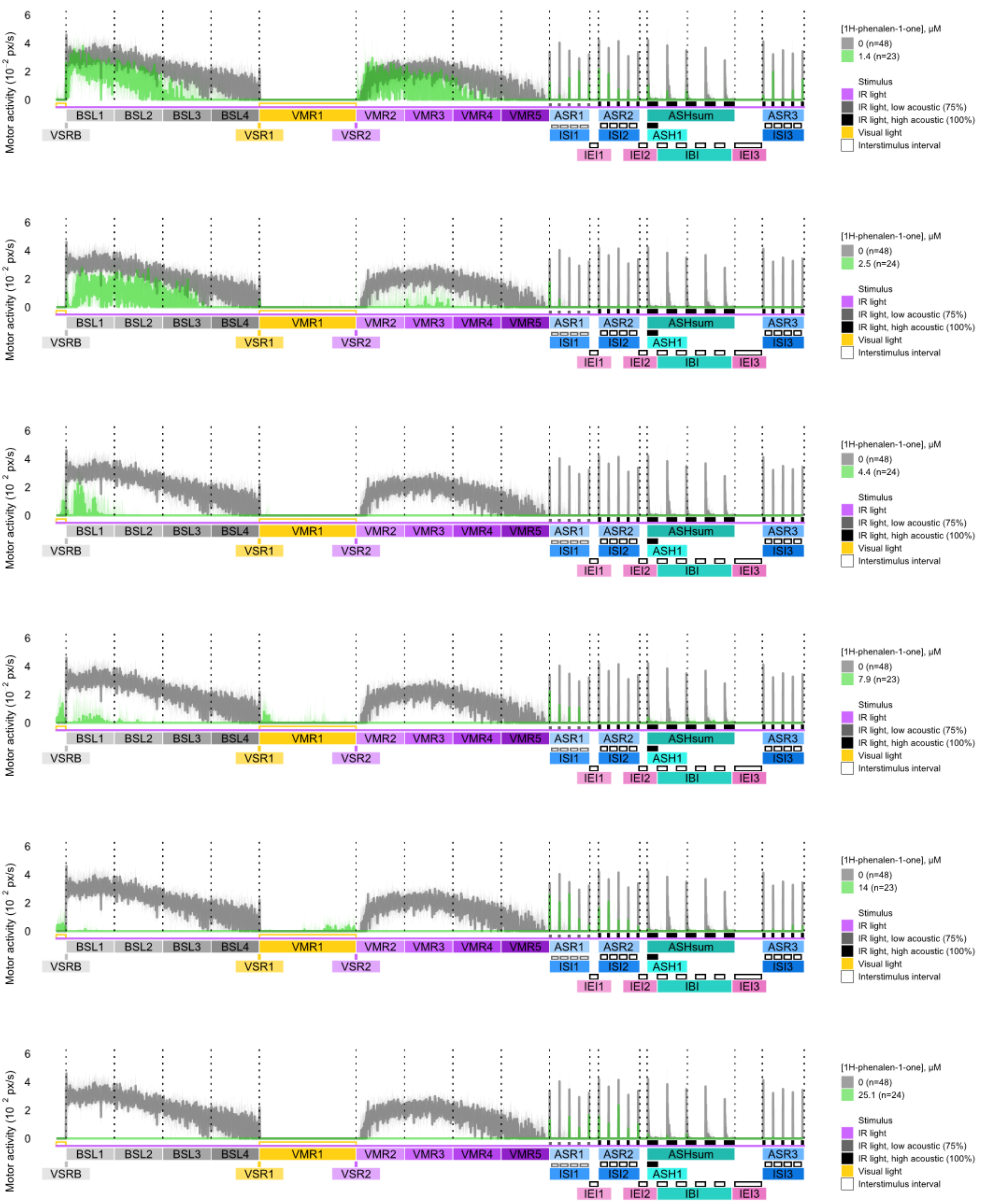

**c 1H-phenalen-1-one**

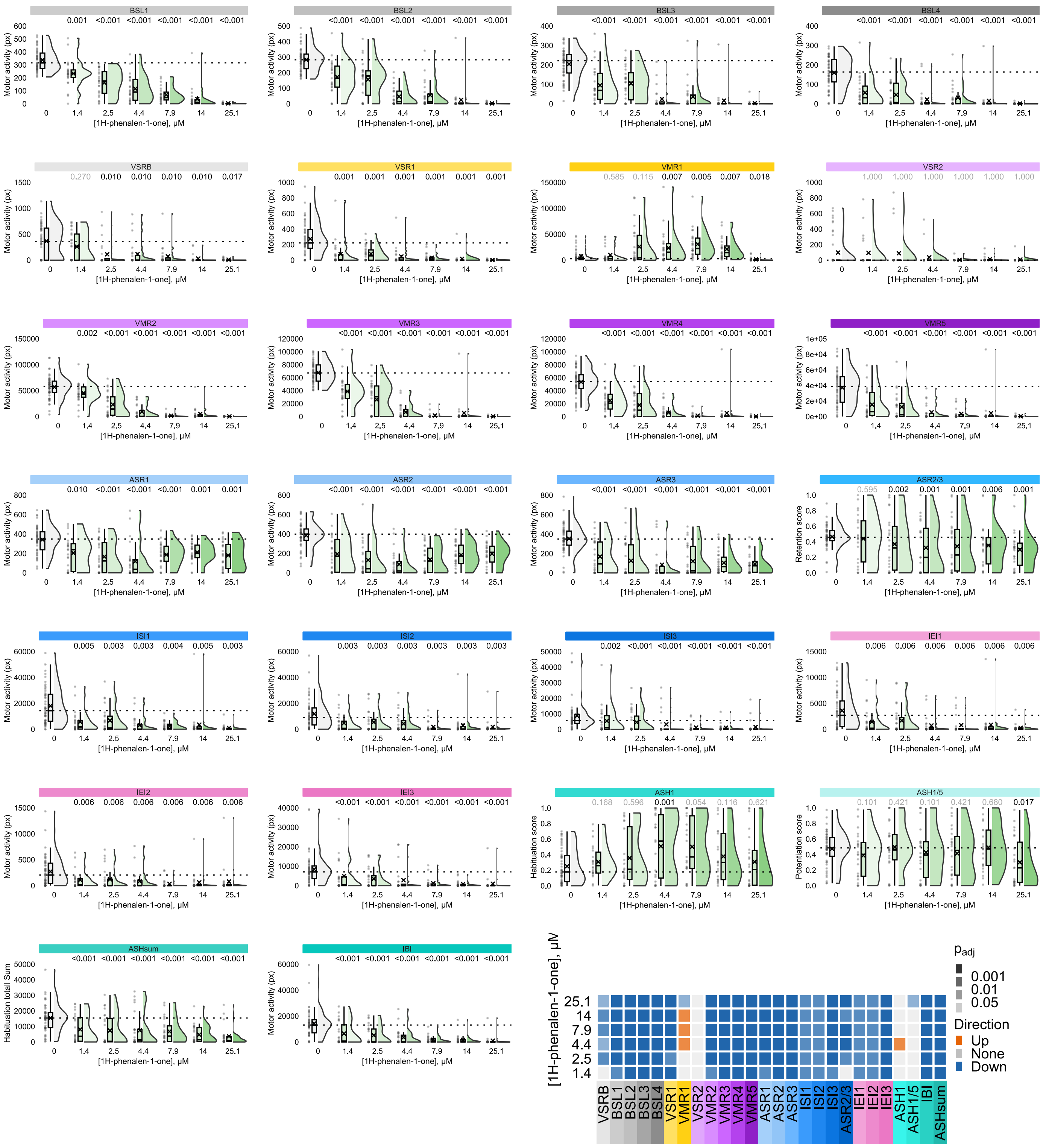

# Figure S7

Motor activity profiles and raincloud plots for zebrafish larvae exposed to the PAH mixture. The y-axis represents motor activity over time, while the x-axis spans 26 behavioral endpoints covering baseline, visual startle, vibrational motor responses, and stimulus-induced activity. Gray traces represent control larvae (n = 40-72), serving as blanks for each respective fraction.

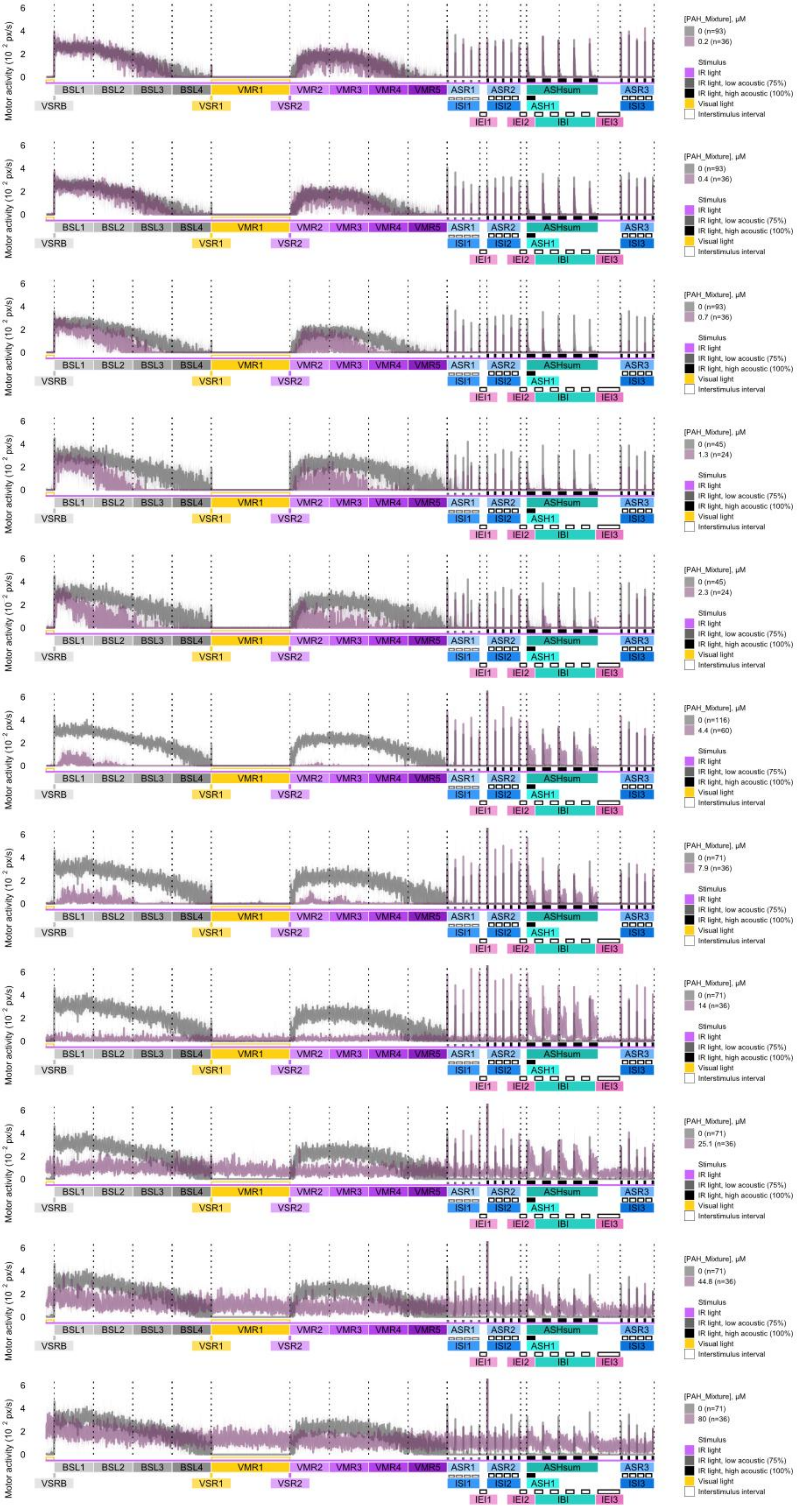

# Figure S7

Raincloud plots of PAH mixture illustrate motor activity differences across the same 26 behavioral endpoints. The horizontal dotted line represents the median control habituation score, and adjusted p-values from a two-sample bootstrapping test are displayed above each plot.

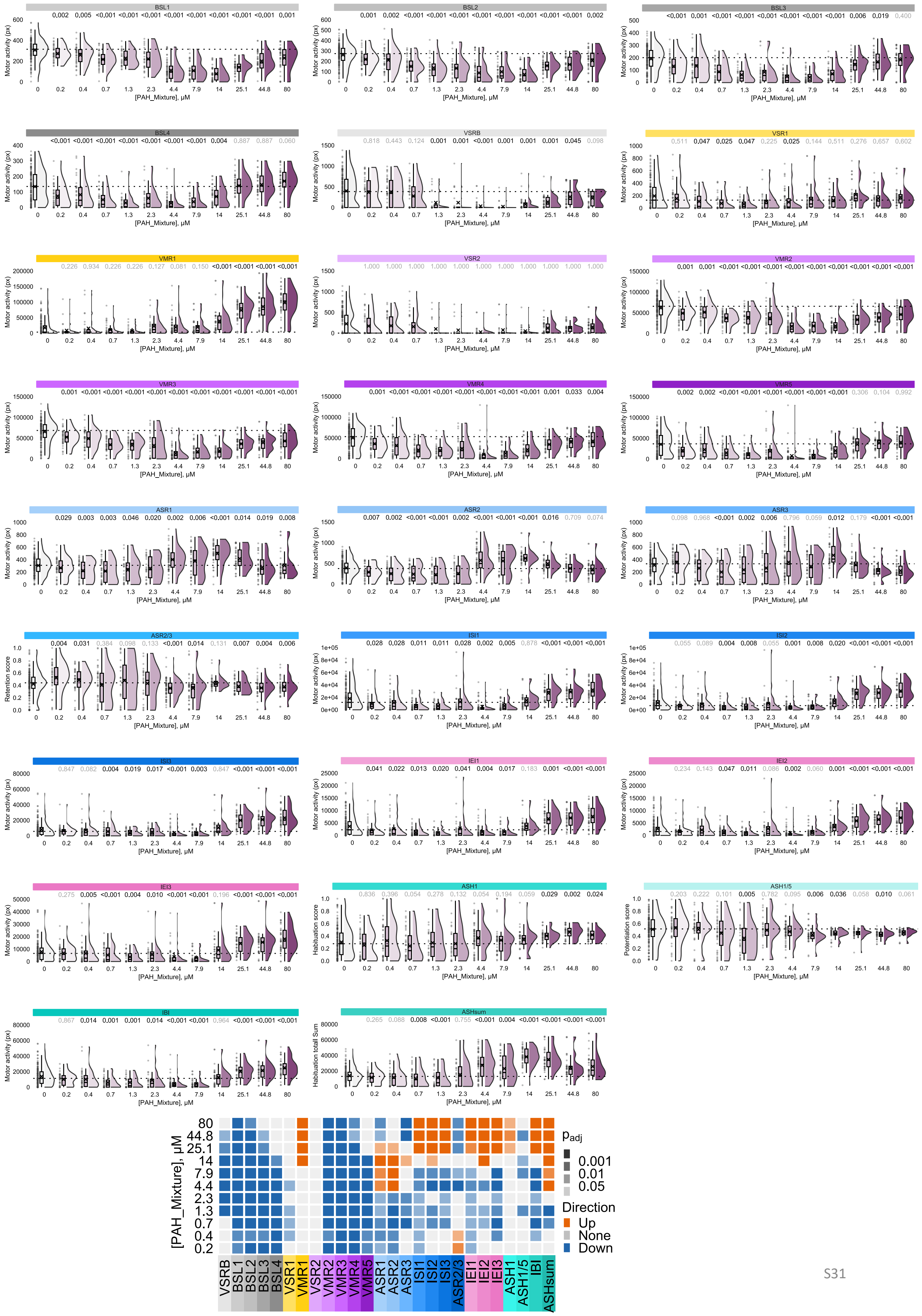

# Figure S8

Hierarchical clustering of behavioral profiles across the 26-endpoint VAMR assay (x-axis), for 63 reference chemicals with known MoAs, obtained from Herold et al. 2025, and the WAF and WAF fractions previously evaluated in this study. Heatmap intensities, based on SSMD effect sizes, are shown with warm colors (orange) indicating increased activity and cool colors (blue/purple) indicating reduced activity relative to the control (n = 36–40 larvae per exposure group).

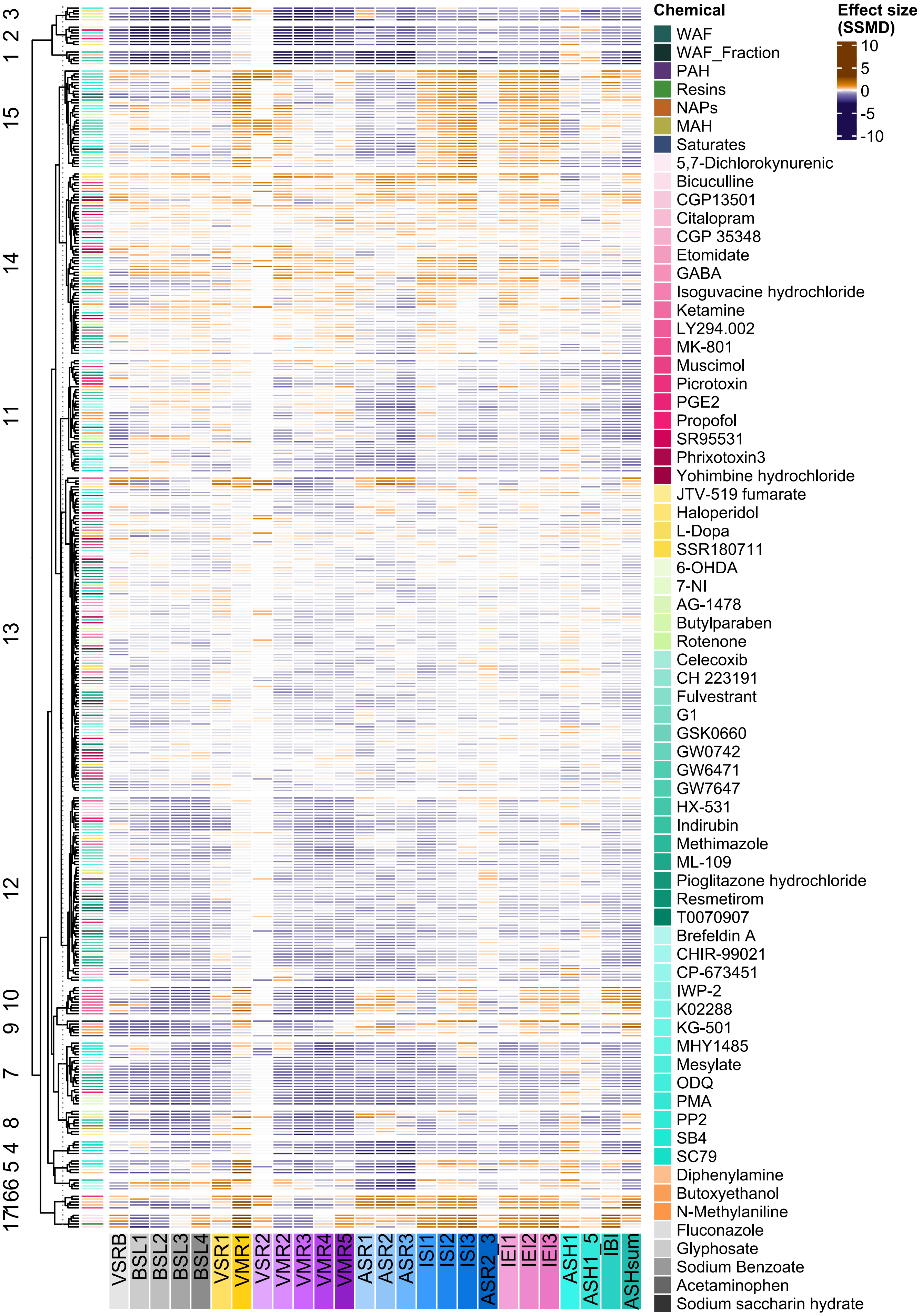

**Table S4 |** Overview of reference chemical clusters associated with neuroactive fingerprinting hits for WAF and isolated fractions

| Cluster | Fraction | Reference chemicals | Target | Function |
|---|---|---|---|---|
| Cluster 8 | NAP & MAH | Butylparaben | Microbial enzyme | Inhibitor |
| | | Haloperidol | Dopamine receptor | Antagonist |
| | | GW7647 | PPARα | Agonist |
| | | JTV-519 fumarate | RyR | Inhibitor |
| Cluster 9 | WAF Fraction & Saturates | Butoxyethanol | Unknown | Predicted non-specific toxicant |
| | | Etomidate | $GABA_AR$ | Agonist |
| Cluster 13 | PAH | ODQ | NO-cGMP | Selective inhibitor |
| | | Bicuculline | $GABA_AR$ | Antagonist |
| | | Haloperidol | Dopamine receptor | Antagonist |
| Cluster 15 | WAF | AG-1478 | EGFR | Inhibition |
| | | Celecoxib | COX-2 | Inhibitor |
| | | CH 223191 | AhR | Antagonist |
| | | GSK0660 | PPARd | Antagonist |
| | | GW0742 | PPARd | Agonist |
| | | HX-531 | RXR | Antagonist |
| | | MHY1485 | mTOR | Activator |
| | | ODQ | NO-cGMP | Selective inhibitor |
| | | SB4 | BMP4 | Agonist |
| Cluster 17 | Resin | Bicuculline | $GABA_AR$ | Antagonist |
